# The Zwicker tone as a model to investigate auditory processing and tinnitus: a scoping review

**DOI:** 10.3389/fnins.2025.1656934

**Published:** 2025-08-22

**Authors:** Jude L. R. Barker, Derek J. Hoare, Magdalena Sereda, Joseph Sollini

**Affiliations:** ^1^Hearing Sciences, Mental Health and Clinical Neurosciences, School of Medicine, University of Nottingham, Nottingham, United Kingdom; ^2^NIHR Nottingham Biomedical Research Centre, Nottingham, United Kingdom; ^3^Department of Speech and Hearing Sciences, University College Cork, Cork, Ireland

**Keywords:** Zwicker tone, auditory after-image, tinnitus, notched noise, Zwicker tone review

## Abstract

The Zwicker tone (ZT) is an auditory illusion experienced by about 50% of the population immediately following a presentation of notched noise (NN). It is a faint, quickly decaying pure tone, the frequency of which falls within the range of the notch. Interestingly, although only half of the general population can perceive ZTs, one study has shown that almost everyone with tinnitus can perceive them. If there is this strong association, the ZT is an easily controllable paradigm that can be employed in the laboratory setting to explore tinnitus related concepts and better interrogate underlying neural mechanisms. This scoping review aimed to catalogue what is currently known about the ZT, and what can be said about its potential relationship with tinnitus. Through a systematic search of the literature, 16 records were identified for inclusion; all reported investigations of ZTs arising after spectrally contrasted in adults who had either normal hearing or tinnitus with/without hearing loss. The proportion of a given sample who were able to hear ZTs varied across studies, from 30 to 100%. The probability of hearing a ZT is modulated by a range of parameters, including: stimulus type (e.g., low-pass versus notched noise), notch centre frequency/width, and stimulus duration. Although these factors modulate the probability of perceiving a ZT, the idea that ZT perception is largely binary is also somewhat supported by individual data. In addition, some variables alter the quality of the percept, e.g., louder stimuli induce a higher pitched ZT. Despite several records drawing comparison between ZT and tinnitus, only one study has thus far investigated ZT in people with tinnitus, albeit finding a highly significant difference in responder rate in tinnitus versus control participants. Several methodological issues potentially affecting responder rate were identified, however, which warrants replication and extension, with careful control. We conclude that (1) Zwicker tone perception relies on a range of factors both stimulus and cognitive related, (2) Further work is required to map the parameters that induce the Zwicker tone and (3) While obvious similarities exist linking tinnitus and Zwicker tones more work is needed to prove the link between the two.

## Introduction

Tinnitus is a condition characterised by the perception of a sound in the absence of an external stimulus. It is a relatively common affliction, affecting around 14% of the adult population, increasing to 24% in adults over the age of 65 (Jarach et al., 2022), and can be a source of great distress to the afflicted (Pinto et al., 2014). Hearing loss and cochlear damage lead to deafferentation which is widely believed to cause, or at least contribute to, tinnitus development (Langguth et al., 2013). The pitch of the tinnitus percept frequently falls within the frequency range of the hearing loss with some evidence suggesting that tinnitus pitch is correlated with the audiometric edge frequency, however these reports are inconsistent (Jain et al., 2021), potentially depending on the specific characteristics of an individual’s tinnitus and hearing loss, reflecting the heterogenous nature of the condition.

It is possible to mimic auditory deafferentation in people who have normal hearing within a laboratory setting using broad band noise with spectral power removed from a specific frequency range, a stimulus referred to as notched noise. In 1964, Ebehard Zwicker reported that notched noise presentation could induce an illusory tone upon stimulus cessation. This auditory aftereffect has subsequently been reliably reproduced many times and since labelled the ‘Zwicker tone’. The Zwicker tone (ZT) is an illusory auditory percept reportedly experienced by about 50% of the population following NN stimulation (DeGuzman, 2012; Parra and Pearlmutter, 2007; Qi et al., 2022; Ueberfuhr et al., 2017). The ZT illusion is perceived as a faint, quickly decaying pure tone, the frequency of which falls within the range of the notch. Its characteristics - frequency and loudness, for example - can be altered by modulating the parameters of the inducing notched noise stimulus, such as the central frequency of the notch and its width.

It is reported that the perception rate of the ZT, ZT prevalence, increases to 95% in patients with tinnitus, more than twice the 42% prevalence reported for the control group in the same study (Parra and Pearlmutter, 2007). The shared illusory nature of tinnitus and ZT, and their association with spectrally contrasted auditory stimuli, contribute to the intriguing nature of the finding, and several subsequent publications have spent time postulating on their relationship (Franosch et al., 2003; Norena et al., 1999; Schilling et al., 2021).

The ZT presents an exciting opportunity to investigate the nature of perception. Furthermore, if indeed tinnitus and the ZT are related, the latter potentially resembles an easily controllable paradigm that can be employed in the laboratory setting to explore tinnitus related concepts and better interrogate the neural mechanisms that may contribute to the condition. There is variation in some of the reported data surrounding ZT, not least its prevalence in the normal hearing population. As such, in this scoping review we set out to catalogue the scientific literature regarding the ZT. Subsequently, the question that motivated this study was: “What is currently known about the ZT and what can be said about its potential relationship with tinnitus?”

## Methods

### Eligibility criteria

Appropriate studies were identified on the basis of predetermined inclusion and exclusion criteria, designed using the PICOS framework, as listed in Table 1.

**Table 1:** Inclusion and exclusion criteria specifying the characteristics that studies must possess to be considered for this review.

Category	Inclusion	Exclusion
Population	Human literature No reported hearing loss OR chronic (>3 months), subjective tinnitus, with or without hearing loss	Pulsatile, objective, transient, or acute tinnitus or tinnitus arising as a treatment side effect Exclusively animal data
Intervention/Exposure	Auditory stimuli containing at least one spectral edge	
Comparator/Context	Investigates Zwicker tone / tonal auditory afterimage arising after spectrally contrasted stimuli presentation	
Outcome	Reports original, human data	
Study Characteristics	Published in English	Abstract or full text unavailable Review articles Discussion or opinion papers, or papers only proposing mechanisms conceptually Computational papers that do not report any new, original data

### Search strategy

We performed the literature search using three databases: PubMed, Web of Science and Scopus. All terms were searched for within the title, abstract and keywords field and were structured as follows:

**Table:** 

(“Zwicker tone”	OR	((“auditory OR “hearing”)	AND	(“afterimage” OR “after image” OR “after-image”)))

Hand searching was also carried out following screening. Reference lists of included records were combed to identify any studies that may have been omitted by our initial search strategy. The abstracts of identified studies were read, and if deemed potentially suitable were added straight to the full-text screening stage.

### Source selection

After removal of duplicates, all retrieved records were screened independently by two reviewers (JLRB and JAS), using the predetermined inclusion and exclusion criteria. Screening was performed using Covidence software. Any discrepancies during independent screening were resolved via discussion and consensus between the two reviewers. Initial screening was performed based on title and abstract, after which full texts were retrieved for remaining references and then rescreened. Exclusion of papers at the full text screening stage required justification based on eligibility criteria, provided by both reviewers independently. Discrepancy either in the full-text inclusion verdict, or exclusion reasoning were again resolved through discussion and consensus.

### Data extraction

The table used for data extraction was designed collaboratively via independent trialling, comparison and consensus by two reviewers. Upon satisfaction with the table and approach, data extraction was performed by one reviewer. During data extraction, snowballing was performed: reference lists of included papers were hand-searched for any other potentially relevant citations not captured by the initial search strategy. Any that appeared suitable upon reading their abstract were added straight to the full-text screening stage to be assessed for inclusion. Where graphical data was extracted, the ‘Plot Digitizer’ tool (https://plotdigitizer.com/app) was used to aid precision. Where notch widths were converted to equivalent rectangular bandwidths (ERBs) the following equation was used: ERBs = 21.4 log10 (0.00437·f + 1), where f represents frequency (Moore and Glasberg, 1983).

## Results

### Search results

**Figure 1:**
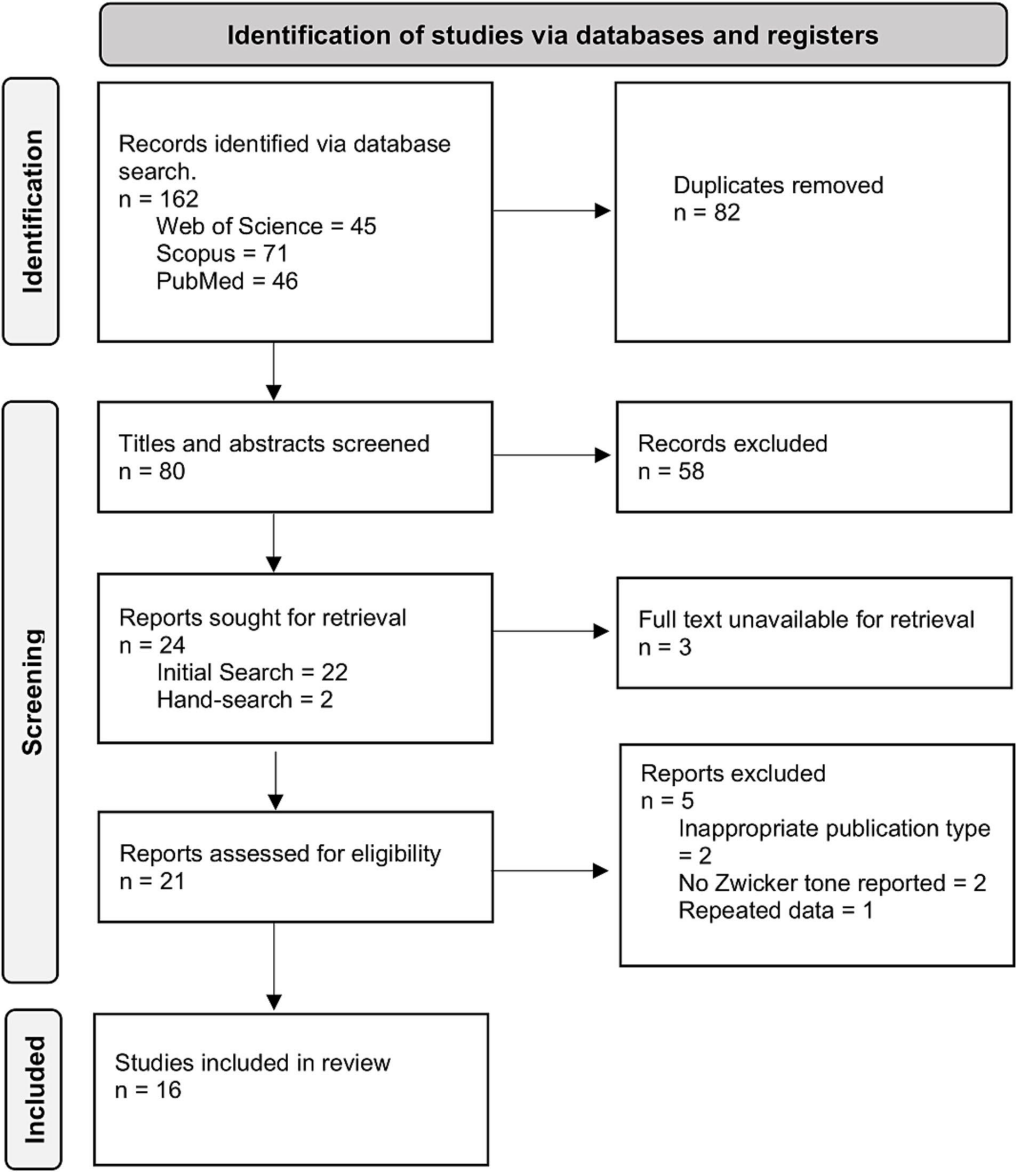
PRISMA flow diagram outlining the process through which 16 records were identified for inclusion.

Following our search strategy we retrieved 162 records. After removal of duplicates, 80 records underwent title/abstract screening, from which 22 papers were identified for full-text screening. Seven papers were excluded having not fulfilled the eligibility criteria, including three for which full texts could not be retrieved. 15 records remained of which one was re-reporting data from another included study and was subsequently also excluded. Hand searching during data extraction identified two more eligible studies, giving a total of 16 records (Figure 1). It should be noted that one included record (DeGuzman, 2012) was a master’s thesis rather than a peer-reviewed paper.

## Study characteristics

### Participants

All records bar one (Zwicker, 1964) reported the age of their subjects to some extent; with mean ages consistently falling between 20 and 30 years old, though some ranges include subjects up to 50 years old (see Table 2). The majority of studies (86%) made some allusion to the hearing thresholds of their participants, with nine reporting audiograms having been performed. Where hearing condition was reported, all subjects were of normal hearing according to the threshold determined by the authors, though said definition ranged from <10 dB 250–8000 Hz to self-reported assessment.

**Table 2:** Participant demographics for those studies reaching the inclusion/exclusion criteria.

Publication			Population			
Author(s)	Year	Title	*n*	*n* Male	Age (mean ± SD; range)	Population/group details
Chen et al.	2025	Auditory illusory models as proxies to investigate bottom-up and top-down neural networks of phantom perception	37	11	ZT high: 22.1 ZT low: 20.5 (Extracted graphical data)	All normal hearing <30dB HL at each frequency 0.5, 1, 2, 3, 4, 6, 8 kHz
DeGuzman	2012	Neural Correlates of Phantom Auditory Perception	24	16	27±4	All frequencies <25dB 0.25–13.75kHz
Fastl and Stoll	1979	Scaling of Pitch Strength	10	Not reported	25–34	"normally hearing"
Fastl et al.	2001	Zwicker-tones for pure tone plus bandlimited noise	9	No info	25–33 (median: 29)	'normal hearing ability'
Gockel and Carlyon	2016	On Zwicker tones and musical pitch in the likely absence of phase locking corresponding to pitch	4	1 (25%)	20–35	Normal hearing, musically trained, no perfect pitch
Hoke et al.	1998	Auditory afterimage: tonotopic representation in the auditory cortex	10	5 (50%)	25 ± 3	Audiogram: <10dB 250-8000Hz All ZT+ and can discriminate between the pitch thereof
Leske et al.	2014	The strength of alpha and beta oscillations parametrically scale with the strength of an illusory auditory percept	12	3 (25%)	24	Normal hearing, audiogram performed
Lummis and Guttman	1972	Exploratory Studies of Zwicker's "Negative Afterimage" in Hearing	28	19 (68%)	20–50	Self-reported absence of auditory defect
Mohan et al.	2020	Investigating functional changes in the brain to intermittently induced auditory illusions and its relevance to chronic tinnitus	47 ZT+:9 ZT-:7	17 (36%) ZT+: 2 (22%) ZT−: 1 (14%)	20.82 ± 2.60 ZT+:20.33 ± 3.57 ZT: 20.71 ± 2.43	Audiogram: <30dB 250–8000 Hz
Noreña et al.	2002	Loudness changed associated with the perception of an auditory after-image	15	7 (47%)	24.8; 20–50	<20dB 500–8000 Hz All ZT+ in response to 1s stimulus
Noreña et al.	2000	An auditory negative after-image as a human model of tinnitus	10	5 (50%)	25.6, 20–30	Audiogram <20dB 500–8000 Hz
Parra and Pearlmutter	2007	Illusory percepts from auditory adaptation	44 TI: 11 NH:33	22 (50%)	28 ± 8	Tinnitus self-reported
Qi et al.	2022	Evidence for predictions established by phantom sound	269 ZT^+^:31 ZT^-^: 20	26 (51%)	ZT+:22.74 ± 2.02 ZT−: 23.40 ± 3.10	Normal audiogram (<25dB)
Ueberfuhr et al.	2017	Modulation of auditory percepts by transcutaneous electrical stimulation	42 ZT+:22	ZT+:7 (32%)	ZT+: 22.4, 18–29	Audiogram: <25dB 250–8000 Hz
Wiegrebe et al.	1996	Auditory enhancement at the absolute threshold of hearing and its relationship to the Zwicker tone	5	Not reported	24–30	All normal hearing
Zwicker	1964	"Negative Afterimage" in Hearing	20	Not reported	Not reported	Not reported

### Induction stimuli

**Figure 2:**
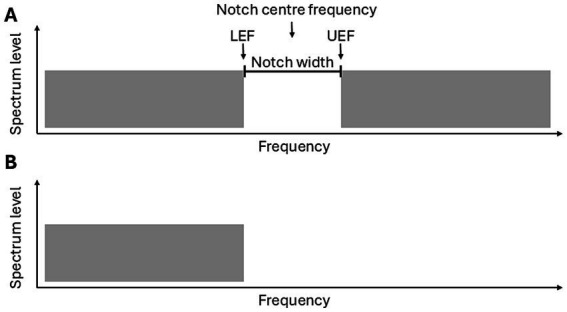
Schematic of the two Zwicker tone inducing stimuli: **(A)** Broadband notched noise and **(B)** Low-pass noise. Important terms, include: the notch centre frequency, LEF = Lower edge frequency (the highest frequency of the lower band), UEF = Upper edge frequency (the lowest frequency of the upper band) and the notch width (the frequency range of the notch).

Stimuli employed were most commonly notched noise, though in some cases low-pass noise was used to induce the percept (see Figure 2; Table 3). Fastl et al. (2001), in addition to low-pass and notched noise, included noise signals with pure tones embedded, either within broadband noise, or at the edge frequency of spectrally contrasted noise which reportedly also generate an illusory tone.

**Table 3:** The stimuli parameters used and summarised outcomes for each study.

Publication			Parameters/Paradigm					Major outcomes/findings
Author(s)	Year	Title	Duration (rise-fall time) /s	SPL/dB	Centre(s) (kHz)	Width(s) (ERBs)	Test/Task	
Chen et al.	2025	Auditory illusory models as proxies to investigate bottom-up and top-down neural networks of phantom perception	3 (not reported)	60	1, 4	1kHz: 5.2 4kHz: 6.1	ZT perception screening followed by EEG	No difference at onset. Significant increase of ERP in response to 4k-NN in ZT+ group vs ZT-, 600-900ms after offset, localised to central-parietal channels. Amplitude increase positively correlated by reported ZT intensity and individual percentage likelihood of perception. Significant increase in theta power 168-296ms after offset during perception, again positively correlated with intensity and percentage likelihood of perception. Source localisation: ZT+ show greater activity in right medial orbitofrontal cortex, right rostral middle frontal gyrus, left lateral occipital sulcus. Reduced activity in right lateral orbitofrontal cortex, right pars orbitalis, left isthum of cingulate gyrus, left lingual gyrus and left pericalcarine cortex.
DeGuzman	2012	Neural Correlates of Phantom Auditory Perception	3 (500ms on, 25 off)	50	0.5, 1, 2, 4	All: 4	Original centres used to identify NN that reliably did/did not produce ZT in each participant Perception rated on VAS from definitely no-unsure-defineitly heard >unsure - yes; <unsure - no EEG performed during stimulus presentation	Comparisons made within groups based on yes vs no responses: No significant difference seen in yes vs no onset responses in any groups Offset responses same yes vs no for ZT- and threshold groups. In ZT+ group, fronto-central negativity seen at 140ms, central-parietal positivity at 340ms (yes vs no sig. diff. p<0.005)
Fastl and Stoll	1979	Scaling of Pitch Strength	60 (Not reported)	47	0.125, 0.25, 0.5, 1, 2	0.125kHz: 3.2 0.25kHz: 3.4 0.5kHz: 3.7 1kHz: 4.1 2kHz: 3.7	Rate pitch strength of ZT /100 vs presented tone	The Zwicker tone has, at its most salient, 100 (very high) pitch strength rating, it strongly resembles a pure tone. Perception prevalence increases with centre frequency, 0% at 125, 30%@250 but variable response. 70% at 500Hz and 100% at 1&2kHz - more reliable responses at 500+
Fastl et al.	2001	Zwicker-tones for pure tone plus bandlimited noise	Not reported	57		Range from 9.7-0.8 Most commonly (each used twice, with/without LEF tone): 2.9@3.7kHz 4.5@4.1kHz 2.6@8.1kHz	24 combinations of NN, BBN, NBN, LPN are combined with pure tones, either at edge frequencies or embedded in the case of BBN. All stimuli presented 4 times in total, in a random order, Particpants respond yes/no and values are assigned by participant 0-4 for each different stimulus	Each combination can induce ZT for some of the subjects, and different subjects respond positively/negatively to different stimuli, with reasonable consistency
Gockel and Carlyon	2016	On Zwicker tones and musical pitch in the likely absence of phase locking corresponding to pitch	5(20ms)	51	2.454, 2.630, 2.818, 3.017, 3.229, 3.456, 3.696, 4.291	2.4kHz:3.4 2.6-3.7kHz:3.5 4.3kHz:3.6	Adjust presented tone pitch and intensity to match ZT or pitch interval below.	All intervals were highly accurate but slightly flat. ZT pitch estimated 1.1-1.2*LEF Monaural presentation may be less salient (still accurate but took longer), left and right could differ from binaural pitch suggesting some integration. Reduced pitch at lower sound level.
Hoke et al.	1998	Auditory afterimage: tonotopic representation in the auditory cortex	3-5s (Not reported) Cycled with 1s silence	45	2, 4, 6	2kHz: 3.7 4kHz:3.9 6kHz:3.4	MEG over left hemisphere during presentation	For all subjects, depth of dipole relative to N1m source during ZT perception increases as ZT pitch increases, suggesting tonotopic representation. Source locations of N1m didn’t differ between stimuli for individuals
Leske et al.	2014	The strength of alpha and beta oscillations parametrically scale with the strength of an illusory auditory percept	1 (Not reported) Cycled with 0.5s silence, AND 5 (Not reported) Cycles with 2s silence	Not Reported	1, 1.414, 2, 2.828, 4, 5.657	0.4-1octaves used as s/m/l 1kHz: 2.1, 3.1, 4.2, 5.2 1.4kHz: 2.2, 3.3, 4.4, 5.5 2kHz: 2.3, 3.5, 4.6, 5.8 2.8kHz: 2.4, 3.6, 4.8, 6.0 4kHz: 2.4, 3.7, 4.9, 6.1 5.7kHz:2.5, 3.7, 5.0, 6.2 0.2-2 octave range to establish best width: 1kHz:1.0, 2.1, 3.1, 4.2, 5.2, 6.3, 7.3, 8.3, 9.4, 10.4 1.4kHz:1.2, 2.2, 3.3, 4.4, 5.5, 6.6, 7.7, 8.8, 9.9, 11.0 2kHz: 1.2, 2.3, 3.5, 4.6, 5.8, 6.9, 8.1, 9.2 10.4, 11.5 2.8kHz:1.2, 2.4, 3.6, 4.8, 6.0, 7.1, 8.3, 9.5, 10.7, 11.9 4kHz: 1.2, 2.4, 3.7, 4.9, 6.1, 7.3, 8.5, 9.7, 10.9, 12.1 5.7kHz:1.2, 2.5, 3.7, 5.0, 6.2, 7.4, 8.7, 9.9, 11.1, 12.3	Best parameters established for each individual to find s/m/l ZT response strengths. (established in 3 stages: yes/no response to NN/WN; 2AFC of different centres; detection curve at best centre for 0-2octave notch widths) EEG performed during ZT perception	Larger widths leads to greater percevied loudness [0.2-1.0 octave range] Increased perceived ZT loudness associated with reduced alpha power, both in increasing notch width, and random perceptual variation within condition. one significant cluster. Reduced power seen 100ms after onset, lasting 400ms
Lummis and Guttman	1972	Exploratory Studies of Zwicker's "Negative Afterimage" in Hearing	4s (5ms) Cycled with 1s silence.	58	2.531	2.5kHz: 4.8	Many parameters are varied in the study HPN is also used	96% ZT+, 37% with HPN. Frequency match 2.1-2.2kHz, similar between NN and HPN. Minimum duration 2s, minimum spectral depth 26dB Addition of upperband of noise doesn't affect the frequency generated by the lower band, rather it extends the range of inducing frequencies. Both noise bands must be presented ipsilaterally to be benefit Addition of tones can alter ZT frequency (pushing frequency of percept away) or obliterate it if too close. Tones presented to the contralateral ear can still affect the pitch of the ZT, though to a lesser extent If different notches are presented to either ear, two distinct percepts can be generated
Mohan et al.	2020	Investigating functional changes in the brain to intermittently induced auditory illusions and its relevance to chronic tinnitus	3 (Not reported)	72	1, 1.414,2, 2.828, 4, 5.657	1kHz: 2.1, 2.6, 3.1, 3.9, 4.2, 5.2 1.4kHz:2.2, 2.8, 3.3, 4.2, 4.4, 5.5 2kHz: 2.3, 2.9, 3.5, 4.3, 4.6, 5.8 2.8kHz:2.4, 3.0, 3.6, 4.5, 4.8, 6.0 4kHz: 2.4, 3.0, 3.7, 4.6, 4.9, 6.1 5.7kHz: 2.5, 3.1, 3.7, 4.6, 5.0, 6.2	ZT perception screening. Reliable ZT+ and ZT- underwent EEG during strong and weak ZT stimuli and BBN	Mean best notch: 0.77octave@3.7kHz (graphical data) ZT+ vs ZT- see significant reduction in ERP ~700-1000ms post ZT stimuli onset; signifcant increase in amplitude ~500-900ms post offset. Significantly greater theta power for ZT+ group, potentially temporal pole, left parietcal cortex and parahippocampus, may be correlated with percept intensiy
Noreña et al.	2002	Loudness changed associated with the perception of an auditory after-image	1 (Not reported)	40	4.043	3	Loudness balancing via 2i-2AFC BBN/NN monaural presentation followed by probe tone ipsilateral and subsequent contralateral probe tone. Report relative loudness of probes	At 5-10dB presentation, tones within the notch perceived as louder following NN vs BBN, outside perceived as quieter. No effect at 20+dB.
Noreña et al.	2000	An auditory negative after-image as a human model of tinnitus	1 (Not reported)	40	4.043	3	2i2AFC, target tone burst	NN sees improved thresholds inside notch, diminished at edge vs BBN, max 2.2 dB. Changes seen in ipsilateral ear. (Report no binaural fusion of ZT, only monaural perception after diotic presentation) LPN see improvement above edge and diminished below edge vs BBN. Benefit lost at higher frequencies
Parra and Pearlmutter	2007	Illusory percepts from auditory adaptation	1000ms rise, 1000ms sustain, 40ms decay	50-60	1.628, 2.306, 3.497	1.6kHz: 17.4 2.3kHz: 13.5 3.5kHz: 9.6	Report any ringing following stimuli, no matter how faint. ZT+ if gave consistent positive reports about any of the NN, but not BBN	Tinnitus sufferers are more likely to perceive ZT: 14/33 NH classed as ZT+, 10/11 TI classed as ZT+.
Qi et al.	2022	Evidence for predictions established by phantom sound	2 (10ms)	60	2, 4, 5.6	2kHz: 3.5, 4.6, 5.8, 6.9 4kHz: 3.7, 4.9, 6.1, 7.3 5.6kHz: 3.7, 4.9, 6.2, 7.4 (s/m/l)	3*NN, 1 WN, 1 WNT (WN followed by tone). For ZT+, WN is oddball; ZT-, WNT is oddball (both 20% likelihood) EEG performed in both attended and unattended conditions	Best notch 1octave at 4kHz (6.1ERB) Attended condition: ZT-dependant MMN and P300 (WN differs from NN for ZT+ group only) WNT deviant for both groups (there is a noticeable perceptual difference vs ZT), but ZT- see larger MMN/P300. Unattended condition: WNT elicits P300 but no MMN, no difference between groups. Theta oscillations greater for ZT+ group Conclusion: ERP components require attention, predictions measured by oscillation don't
Ueberfuhr et al.	2017	Modulation of auditory percepts by transcutaneous electrical stimulation	3 (Not reported) Cycled with 2s silence	50/55	4.043	3	Match subsequently presented tone to loudness and AM depth of the perceived tone Electrical stimulation occurs during silence	Electrical stimulation led to siginificant AM of ZT at 3mA. Similar to how stimulation can also lead to AM of externally presented tones.
Wiegrebe et al.	1996	Auditory enhancement at the absolute threshold of hearing and its relationship to the Zwicker tone	2-3s (Not reported) Varied by individual for best ZT	30, 40	4	4kHz: 3.0, 6.1	Bekesy tracking system vs silence 2AFC vs BBN	Up to 13dB improvement vs in silence at frequencies within the notch. (7.2dB improvement vs BBN by 2AFC, no change outside notch) Maximum threshold improvement seen around ZT pitch, no improvement seen at edge of notch or following BBN. Changing parameters alters ZT pitch and frequency of maximum improvement correspondingly. Mean changes: 3.4-4.8kHz@40dB: 8.4dB 3.4-4.8kHz@30dB: 4.3dB 2.9-5.8kHz@40dB: 5.2dB
Zwicker	1964	"Negative Afterimage" in Hearing	60 (Not reported)	40-70	2.7	2.7kHz: 3.5	Descriptive report and frequency matching	90% ZT+ naïve, 100% with prompting. Generally, percept lasts 5-10s; duration of percept increases with inducer duration Sound level can't be too high or low. Pitch higher at higher intensity and lower at low. The effect is greater at higher frequencies. Notch widths generally 0.5-2octaves, percept is less clear otherwise 5Hz cycled 100ms inducer can produce continuous percept. If different notches are presented to either ear, two distinct percepts can be generated. If continuous noise is presented to one ear, only the offset-ear will perceive the ZT

**Figure 3:**
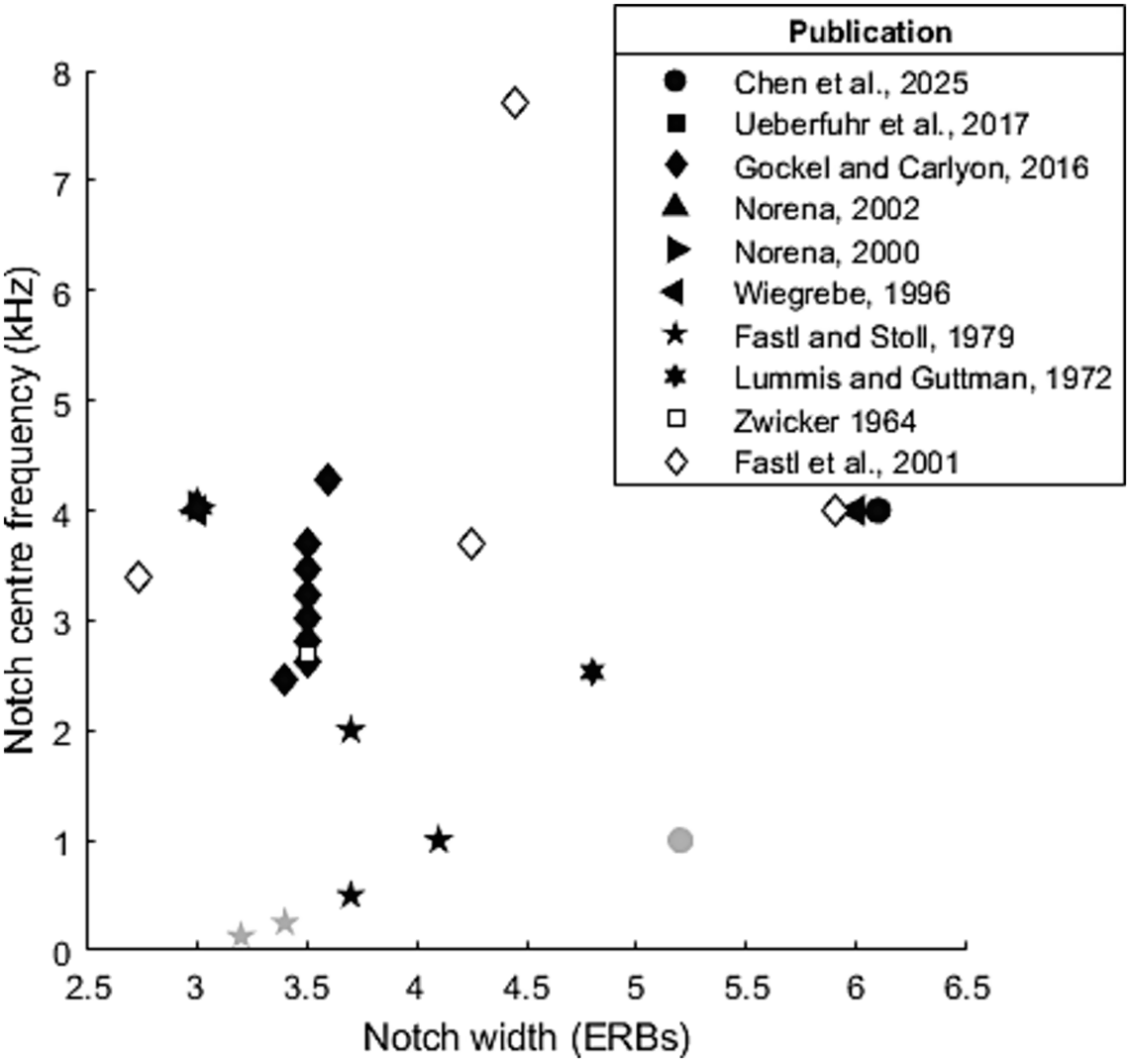
Parameters (notch centre frequency and width) used to test Zwicker tone perception. Black and filled symbols indicate that at least one participant was able reliably perceive a Zwicker tone at this notch frequency/width combination. Grey symbols indicate where no Zwicker tone was reliably induced.

In the case of notched stimuli, the centre frequency used ranged from 500 Hz to 8.1 kHz, though these extreme values were primarily used in studies exploring ideal parameters, with most studies primarily using centre frequencies between 1–6 kHz (see Figure 3). Several studies (Chen et al., 2025; Fastl and Stoll, 1979) have failed to induce a ZT at low notch centre frequencies (250, 500 and 1,000 Hz). While Chen et al. (2025) did not induce a ZT with a 1 kHz centre frequency notch (5.2 ERBs, 3 s), Fastl and Stoll (1979) did (4.1 ERBs, 60s). This likely reflected the increase in noise duration (from 3 to 60s) rather than the modest difference in ERB width. Generally, it seems that the lowest centre frequency at which the Zwicker tone can be induced likely lies between 1 and 2 kHz for short duration noise, though this could be reduced by varying other parameters such as notch width or noise duration.

Reported notch widths range from 0.8–17.4 ERBs, though again these extreme values again appear to be outliers, both only appearing in one paper each, with the majority of widths tending to within 3-7ERBs. Leske et al. (2014) attempted to find the most effective parameters, and their ranges have been re-employed by studies which have subsequently reported the notch frequency/width combination most effective at inducing ZT perception: Qi et al. (2022) report this as being a 1 octave (6.1ERB) notch centred at 4 kHz, while Mohan et al. (2020) it as a 0.77 octave (4.7ERB) notch centred at 3.7 kHz.

In addition to notch width and centre frequency other parameters also appear to modulate the probability of perception and perception strength. Lummis and Guttman (1972) varied spectral-gap depth (the relative spectrum in the notch versus the upper and lower noise bands) and the noise duration. They found that for short duration noise (less than 2–3 s) a large gap-depth (i.e., slope of the notch in the frequency domain) is required becoming increasingly large as duration reduces. Above 2–3 s the gap depth becomes less of a factor and stimuli of most durations/gap depths (above 30 dB) produce a reliable percept. The same authors also suggest that spectrum level of the noise is a factor where 10-30 dB spectrum level represents a sweet spot for inducing the ZT, whereas at higher spectrum levels the percept is less likely to be perceived.

### Perceptual prevalence

There is considerable variation in the reported prevalence (see Table 4) of listeners who do perceive Zwicker tones (from hereon we shall refer to this population as ZT^+^), as opposed to those who do not (ZT^−^). Zwicker (1964) originally stated that 90% of naive subjects could perceive his auditory after-image, with this statistic increasing to 100% upon prompting. Lummis and Guttman (1972) report a similar figure, 96% following notched noise, along with 37% when only high-pass noise is used. However, more recent studies suggest a significantly lower value: ranging between 30 and 52% (Parra and Pearlmutter, 2007; Ueberfuhr et al., 2017; Chen et al., 2025).

**Table 4:** Zwicker tone perception prevalence data (for those studies reporting these data).

Authors	Year	Prevalence	Screening	Binary or Categorical	Method of Assessment
Chen et al.	2025	30% ZT+ 70% ZT−	Y	B	Initial screening of <50% positive (3+ on rating scale) response to WN and 1kHz-NN controls ZT-high: >50% positive (2+) response to 4kHz-NN ZT-low: <50% positive (2+) response to 4kH-NN
DeGuzman	2012	ZT+: 58% Threshold: 17% ZT-: 25%	N	C	Initial testing to establish personalised test and control notches. Accuracy based on % correct responses to NN and control stimuli. 85%+: ZT+ 65–85%: threshold 35–65%: ZT−
Lummis and Guttman	1972	Notched noise: 96% High-pass noise: 37%	N	B	Asked to report whether soft tonal sound was perceived in silence between stimuli.
Mohan et al	2020	ZT+: 20% ZT−: 32%	Y	C	Participants screened on ability to respond positively to NN and negatively to WN Subsequent testing using NN, and WN and NN controls. ZT+ >50% positive response to ZT+ NN ZT- <50% positive response to ZT+ NN NB. Due to study design ZT− group consists of people who could respond to notched noise positively, but not when a negative control was included.
Parra and Pearlmutter	2007	ZT+: NH 42% Tinnitus: 91%	N	B	Presented WN and three NN stimuli Based on consistent responses to 'which of the four noises was followed by a perception of some form of ringing, however, faint it might be.
Qi et al.	2022	17% ZT+ 11% ZT−	Y	C	Initial screening of >90% accuracy to WN and WNT. ZT+: Mean >90% positive response to NN with best centre 4kHz ZT−: Mean <10% positive response to NN
Ueberfuhr et al.	2017	ZT+: 52%	N	B	Subjects reported a stable percept: 12@55dB SPL, 10@50dB SPL.
Zwicker	1964	90% naïve; 100% prompting	N	B	Asked to describe what they heard following stimulus, later prompted if failed to report ZT.

The use of pre-screening before categorisation is reported. (NN - Notched noise (ZT-inducing, unless specified); WN -White Noise; WNT – White noise, followed by pure tone).

While only five studies present ZT prevalence as a binary statistic, i.e., grouping subjects into ZT^+^ and ZT^−^, three further studies instead grouped participants into more than two categories representing the likelihood of perception over a scale. While we cannot confidently convert these categories into an equivalent binary ZT^+^ and ZT^−^ statistic, we can extract the reported ZT^−^ figure (classified in these studies as those individuals least likely to respond positively to ZT stimuli) and from it infer an upper-bound to the ZT^+^ statistic. These values range from 11 to 32%, suggesting a maximum ZT prevalence of between 68 and 89%. Admittedly, this statistic cannot be used to confirm the more recent, lower values of ZT prevalence, but combined with said values they do cast doubt on the very high ZT prevalence originally reported by Zwicker (1964) and Lummis and Guttman (1972). Finally, ZT perception has been repeatedly linked to tinnitus perception but, to date, only one study has empirically tested this potential link. Parra and Pearlmutter (2007) found a prevalence of 91% in individuals with tinnitus compared to 42% in individuals with normal hearing.

In the reported assessments and surveys establishing ZT prevalence, the inclusion of a white noise control stimulus is common, with some records also employing a non-ZT inducing notched noise as a further control. Where ZT prevalence is being calculated, control stimuli are mostly used to pre-screen participants based on a threshold set by the authors, before the remaining participants are organised based on their responses to notched noise (Qi et al., 2022; Chen et al., 2025; Mohan et al., 2020). The thresholds for exclusion in these studies vary between 50–90% correct rejection of the white noise. However, DeGuzman (2012), determined perception based on accuracy calculated using both hits (responding positively to ZT inducing stimuli) and correct rejection.

### Percept frequency

Zwicker’s original observation that the frequency of the percept falls within the range of the notch of the inducing stimulus has been consistently reproduced in situations where notched noise is employed. In support of this, five studies report data from frequency matching tasks in which a quiet tone is adjusted to match the perceived pitch of the Zwicker tone. Gockel and Carlyon (2016) having investigated ZT-pitch matching in musically trained individuals, finding that, as a general rule, the frequency of the percept is roughly 1.1–1.2 times the frequency of the lower edge of the notch (i.e., the edge frequency of the low frequency noise band). Interestingly, another study reported ZT^+^ participants perceive the Zwicker tone following low-pass noise and the addition of an upper noise band has little effect on frequency (Lummis and Guttman, 1972). This suggests that the pitch of the Zwicker tone is strongly influenced by the edge frequency of the lower band of noise. However, while the higher noise band does not seem to alter the ZT pitch an increase in notch width, and hence a higher upper edge frequency, seems to result in a wider range of notch widths that produce a ZT (Lummis and Guttman, 1972) and greater perceived ZT loudness (Leske et al., 2014; Lummis and Guttman, 1972).

**Figure 4:**
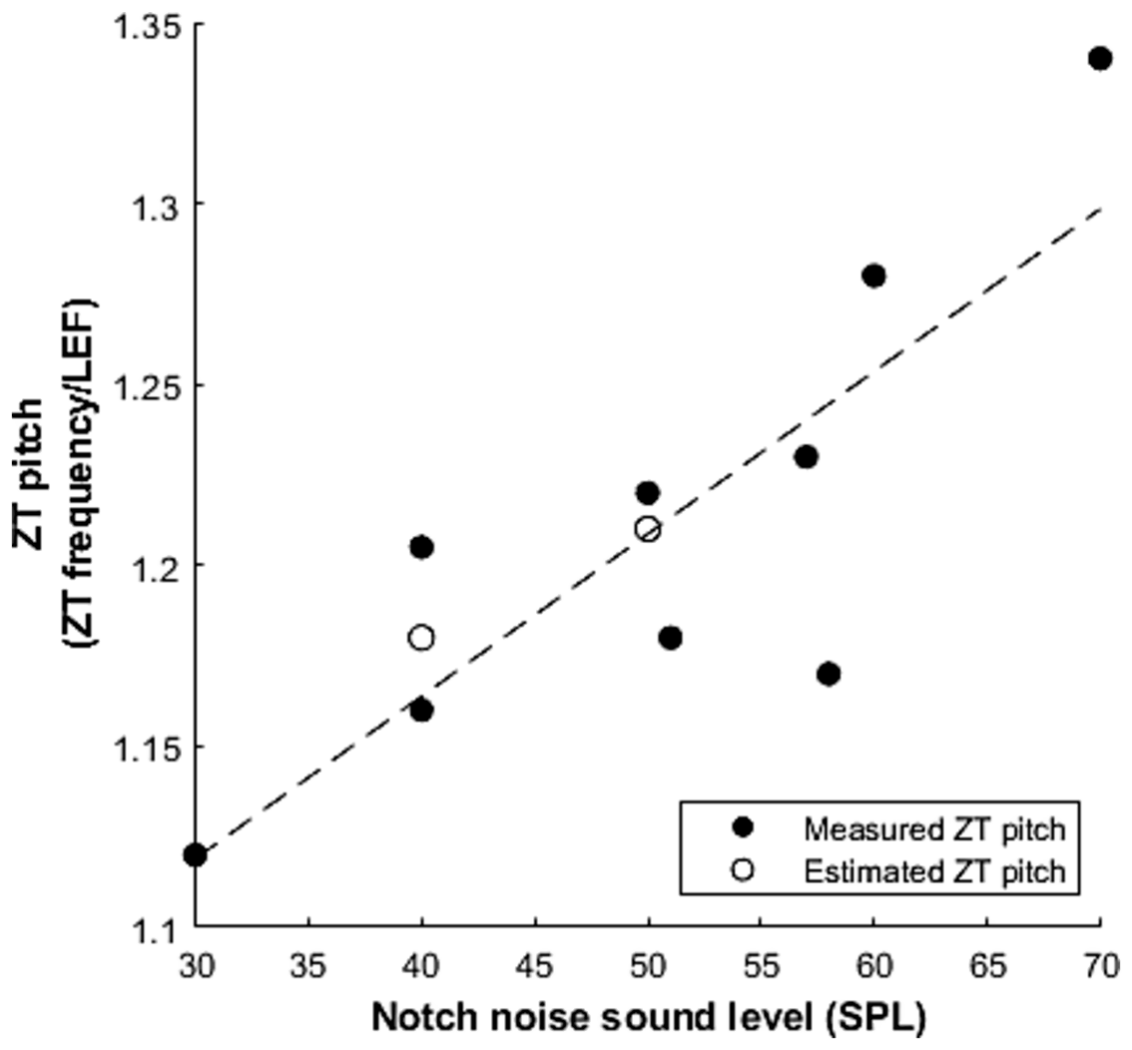
The linear relationship between the measured ZT pitch and the sound level of the notched noise (r (7) = 0.83, *p* = 0.006). ZT pitch is converted into a ratio of the reported ZT frequency over the lower edge frequency (LEF) of the noise. Filled circles represent data from pitch matching experiments (Lummis and Guttman, 1972; Fastl et al., 2001; Gockel and Carlyon, 2016; Wiegrebe et al., 1996; Zwicker, 1964) and empty circles represent an estimate of Zwicker tone pitch based on experiments measuring threshold changes in the notch (Alves- Pinto and Lopez- Poveda, 2008; Norena et al., 2000).

Where studies performed frequency matching, we extracted mean values, converted them into ratios of perceived ZT frequency to lower edge frequency (LEF) of the inducing notched noise. This reveals a range of 1.12–1.34 times the lower edge frequency of the inducing notched noise (Table 5). This is a wider range than predicted by Gockel and Carlyon (2016), however some variation in the ratio should be expected not least because, as previously stated, the intensity of the inducing stimuli is a known factor: higher SPL stimuli induce higher pitched Zwicker tones. Interestingly, both Zwicker (1964) and Wiegrebe et al. (1996) explore the effect of increasing SPL and report a similar increase in ZT pitch (ZT/LEF ratio) as a function of SPL: +0.06/10 dB and +0.10/10 dB increase, respectively. To explore this relationship between percept frequency and sound level, we plotted our extracted ZT pitch as a function of SPL, revealing a linear positive correlation across the data points from these studies (r (7) = 0.83, *p* = 0.006, Figure 4). Furthermore, when considering the lower edge frequency as the informant of the percept frequency, Wiegrebe et al. (1996) varied both the notch width and intensity and found a much greater effect of intensity than notch width on the frequency multiplication factor.

**Table 5:** ZT-lower edge frequency ratio and noise presentation level (SLPdB).

Authors	Year	SPL(s)/dB	Mean ZT frequency/kHz	Lower Edge Frequency/kHz	ZT-LEF Ratio	
Fastl et al. *	2001	57	3.87	3.15	1.23	
Gockel and Carlyon *	2016	51	2.27	2.0	1.14	Mean = 1.18
			2.62	2.14	1.22	
			2.72	2.30	1.18	
			2.87	2.46	1.17	
			3.06	2.63	1.16	
			3.38	2.82	1.20	
			3.63	3.10	1.17	
			4.10	3.50	1.17	
Lummis and Guttman	1972	58	2.22	1.90	1.17	
Wiegrebe et al.	1996	40	4.14	3.40	1.22	Mean = 1.205
			3.45	2.90	1.19	
		30	3.8	3.40	1.12	
Zwicker	1964	40	2.55	2.20	1.16	
		50	2.7		1.22	
		60	2.82		1.28	
		70	2.95		1.34	

Where ZT frequencies are estimated for multiple notches at the same sound level within a single study, a mean ratio has been calculated for each sound level. Data from those records marked with an asterisk (*) were extracted from a graphical source via plot digitiser.

### Threshold/loudness changes

Three papers report changes in perception of tones presented following notched noise in ZT^+^ participants. Presentation of a notched, ZT-inducing noise precursor can reduce detection threshold for a pure tone presented just after noise offset, both versus a broadband (ie. without a notch) noise precursor (Wiegrebe et al., 1996; Norena et al., 2000), and versus detection in silence (Wiegrebe et al., 1996). The reported magnitude of this effect varies: a maximum mean improvement of 8.1 dB is reported for vs. silence (Wiegrebe et al., 1996), while improvements vs. broadband noise range from 2.2–7.2 dB (Wiegrebe et al., 1996; Norena et al., 2000) – all at 40 dB SPL noise presentation. In addition to notched noise, low-pass noise (also ZT-inducing) sees a similar though lesser effect versus broad band noise (Norena et al., 2000). Interestingly, detection thresholds are reduced at frequencies within the notch, but for those frequencies proximal to the spectral edges of the noise an increase in thresholds is reported (Norena et al., 2000). Again, a similar effect is reported for low-pass noise, with an increase in detection threshold at the noise edge and a reduction in threshold for tones above the edge frequency. This benefit to detection threshold is then lost at higher frequencies, returning to baseline as the frequency becomes more distant from the noise edge (Norena et al., 2000).

As with ZT perception, detection threshold improvements vs. silence are modulated by the width of the notch and the level of the noise, with wider notches and quieter noise presentation reportedly resulting in lesser threshold reductions (Wiegrebe et al., 1996). However, only one comparison has been made of each: 30–40 dB and 3–6.1ERBs, and as such this relationship may vary over greater ranges, likely peaking at some value for either parameter before reducing again rather than improving indefinitely. One study also reported ZT frequency matching and found that maximum threshold improvement to be similar to the reported frequency of the ZT (Wiegrebe et al., 1996). Furthermore, Norena et al. (2000) report threshold improvement exclusively in the ear ipsilateral to ZT perception suggesting the two phenomena may be related. However, all participants in said study reportedly experience a monaural percept, in contrast to previous findings that suggest binaural fusion of the percept after binaural presentation (Lummis and Guttman, 1972).

Finally, in addition to changes in absolute thresholds, perceived loudness of a subsequently presented pure tone is also reportedly coincidental with ZT perception at low presentation levels (5–10 dB above absolute threshold), with ipsilaterally presented tones perceived as louder following notched vs. broadband noise (Norena et al., 2002). Again, this effect is seen at a frequency within the notch, while the opposite effect is seen outside of the notch with tones perceived as quieter. At higher presentation levels (>10 dB above threshold), no difference is reported in perceived loudness either within or outside of the notch.

### Cortical electrophysiology studies

We identified six records that report data from cortical electrophysiology studies (five EEG and one MEG), and various neural correlates of Zwicker tones are presented. The heterogeneity of methods, stimuli and results make comparison complicated, though below we attempt to synthesise these studies. That said, the experimental designs used can be, broadly, categorised into two groups. (1) within-subject designs: trials in which a participant responded positively to a stimulus are compared to those trials in which they do not report ZT perception. (2) between-subject designs: trials are compared between ZT^+^ and ZT^−^ groups for ZT-inducing and non-inducing stimuli.

### Evoked response potentials

An increase in ERP amplitude is reported in ZT^+^ subjects vs. ZT^−^ following ZT inducing stimuli (between-subject design), around 500-900 ms post noise offset (Chen et al., 2025; Mohan et al., 2020). This result has been seen both where the ZT-inducing stimulus was homogenous (Chen et al., 2025) and bespoke (Mohan et al., 2020) for each subject. Conversely, when using a within-subject design, DeGuzman (2012) found much earlier ERP differences, one around 140 ms and one around 340 ms when ZT^+^ participants responded positively to ZT-inducing stimuli vs. negatively. These differences were not observed in ZT^−^ subjects, i.e., when comparing their ZT positive trials (responded yes to a ZT) versus negative trials. Chen et al. (2025) also observed a significant correlation between evoked ERP amplitude and both the probability of perceiving a ZT and subjective ZT intensity.

### Oscillatory power

Oscillatory power within the theta band (3-8 Hz, as defined by the included studies) has been shown to be increased generally (i.e., total theta power) and following ZT-inducing stimuli (i.e., induced theta power) in ZT^+^ vs. ZT^−^ subjects (Qi et al., 2022; Chen et al., 2025; Mohan et al., 2020). Of these, two studies found an increase in induced theta between 200 and 900 ms (Qi et al., 2022; Mohan et al., 2020). Whereas, Chen et al. (2025) found an increase in induced theta power between 168 and 296 ms but not during the later 600 and 900 ms epoch. Average total theta power (averaged across significant channels during the significant epoch) scales significantly with ZT percept strength (Chen et al., 2025; Mohan et al., 2020) and the probability of perceiving a ZT (Chen et al., 2025). In addition, single-trial theta power also significantly predicts the ZT percept strength (Mohan et al., 2020).

Alternatively, Leske et al. (2014) found reduced oscillatory power in the alpha/beta range (10-20 Hz) when contrasting neural responses to ZT inducing vs. non-ZT inducing stimuli (within-subjects design) in ZT^+^ subjects. As with the above studies this neural correlate was found to scale significantly with the strength (loudness) of the ZT (Leske et al., 2014). Meanwhile, DeGuzman (2012) did not report a significant effect of post-stimulus offset (ZT related) alpha and did not find a significant change in ongoing alpha (during the notch noise) in ZT^+^ subjects. However, they did observe an increase in frontal alpha on trials in which ZT^−^ subjects responded positively, reporting ZT-perception, during the ongoing stimulus.

### Neural sources

Source localization of ERPs does not appear to be consistent across this limited number of studies (three records). Mohan et al. (2020) suggest sources in the temporo-parietal junction, right parietal and auditory cortex. Whereas, the same group subsequently found increased activation in right medial orbitofrontal cortex, right rostral middle frontal gyrus and left lateral occipital sulcus (Chen et al., 2025). In addition, DeGuzman (2012) observed an early negativity (~140 ms) on fronto-central channels and the later positivity (~340 ms) on central-parietal channels. Neural correlates of the ZT have been reported as represented tonotopically, with equivalent current dipole depth increasing with ZT frequency (Hoke et al., 1998), though this has not been replicated. Source localisation of theta power has, simialrly to ERP source localization, been inconsistent across studies. Interestingly, the ZT percept can be amplitude modulated by transcutaneous electrical stimulation, similar to real tones (Ueberfuhr et al., 2017).

## Discussion

To the best of our knowledge, this is the first study to catalogue all available human Zwicker tone literature. While the literature describing Zwicker tone perception is relatively small a number of observations can be made.

### Prevalence

Our results show great variation in the reported prevalence of ZT perception, ranging from 30–100% when presented as a binary statistic. As stated in our results, in cases where perception is categorised into more than two groups, using the ZT^−^ statistic to infer an upper limit of ZT prevalence suggests that Zwicker (1964) and Lummis and Guttman (1972) estimates of 96–100% may be too high and more conservative estimates may be more accurate. A range of possible factors might explain the differences in observed ZT prevalence.

For example, the stimuli used may have an influence on the prevalence observed. Zwicker (1964) used 60s noise bursts to induce the ZT, far longer than the standard 2–3 s duration utilised in the literature. Lummis and Guttman (1972) employed a more typical signal duration of 4 s, however this was cycled with 1 s off durations and the majority of subjects reported ZT perception within 2 min, though some only weakly. In contrast, Ueberfuhr et al. (2017) report a perception probability of only 52% having used a 2 s on-off cycle: they do not report maximum lengths of time allowed for participants to potentially perceive the ZT, but they do state that the stimulus sequence could be restarted at any time. It may be that the ZT is elusive for many people and initially unperceivable, but extended presentation allows the percept to appear, albeit faintly, however more recent uses of extended, cycled stimuli suggest this may not be the sole cause for increased perception probability.

The participants themselves may present a factor in this disparity: Zwicker (1964) omits any demographic information about his subjects, though it is not unreasonable to suspect that they may well have been lab colleagues. Meanwhile, Lummis and Guttman (1972) state that their initial survey was completed by ‘randomly selected laboratory personnel, most of whom were naive listeners’. Though said listeners were reportedly naïve, the distinction between lay people and auditory laboratory personnel may well be important, the latter presenting a group of potential expert listeners.

Finally, the methodology of these earlier papers is potentially less robust than more recent studies that include control conditions and false alarms: rather they simply ask whether a percept is experienced or ask participants to describe their perception. The lack of controls make it difficult to assess whether participants were indeed detecting the tone reliably, though the inclusion of frequency matching provides some confidence in the findings. Furthermore, the question used to probe whether listeners heard the percept - i.e. asking listeners whether they heard a soft tonal sound between noise bursts - while maintaining naivety regarding the source of the percept (indeed, participants are reportedly surprised to learn that the ZT is not externally delivered), does prompt the expectation of a tone, perhaps doubly confounding in the absence of a control condition. Although, it should be noted almost all studies will have informed participants that they are listening for a soft tone after noise cessation, i.e., prompting listeners expectation. There is some support for the idea that prompting/priming might increase ZT prevalence. Zwicker (1964) reported that prevalence increased upon prompting and description of the percept: “For only two listeners was it necessary to discuss in detail the sound that they should listen for. They may have expected too much, and after the discussion they were able to detect and to match the pitch of the subjective sound in the same way as the other 18 people.”

### Parameters for ZT induction and modulating perception

A range of parameters appear to be important for ZT induction. Overall, the general features of the Zwicker tone presented are consistent with Zwicker’s original report – a tonal, illusory percept, experienced after the offset of broad band noise containing a spectral notch or low frequency broadband noise (though a notch is more likely to cause the percept and makes it louder). The centre frequency of the notch and the notch width both also modulate the probability of perception as does the duration of inducing stimuli. When presented monaurally, it is only perceived in the ear ipsilateral to presentation; binaural, diotic presentation leads to central, fused perception; and dichotic presentation leads to two distinct, lateralised percepts. The percept increases in duration with a longer induction stimulus, and a 100 ms inducer presented at a repetition frequency of 5 Hz can produce an ongoing percept. The frequency of the percept falls within the region of the notch, and an increase in inducer sound level leads to an increase in percept frequency, though increasing the sound level too high may reduce or prevent the percept (Lummis and Guttman, 1972). In addition, when calculating percept frequency as a ratio of lower edge frequency and plotting it as a function of sound level, our results support the suggestion that the frequency of the ZT is related to the lower edge frequency of the inducing stimuli. This also suggests that sound level is broadening auditory filers to push the percept higher in frequency.

### Classification of ZT perceivers

The approach to categorising an individual as ZT^+^ or ZT^−^ might also benefit from examination. As noted, the use of control stimuli in the form of white noise or non-ZT inducing notched noise, is common and arguably essential. Indeed, both Zwicker (1964) and Lummis and Guttman (1972) omit a control stimulus in their initial surveys, which may contribute to their higher reported ZT prevalence.

Currently both binary, ZT^+^ vs. ZT^−^, and more continuous categorisation, ZT perception on a scale, are used. The potential difficulty associated with ZT detection, as suggested by the higher prevalence associated with longer noise exposure, may make an argument for a more continuous categorisation, i.e., rating subjects on the likelihood of their perception rather than assigning them a binary label. If indeed the salience of the percept varies between individuals on a continuous scale, it stands to reason that said salience would be similarly continuous as a function of stimulus parameters. Multiple studies have explored the use of a range of parameters in order to idealise stimuli or tailor them to individual participants (Qi et al., 2022; Leske et al., 2014; Mohan et al., 2020), however the distribution of percept rating values has not been reported in detail. Supplementary data from Qi et al. (2022), demonstrates that when given a 0–9 scale, participants tend to respond in a primarily binary fashion, demonstrating a preference for extreme ratings of 0 or 9, suggesting that perception may be closer to a binary effect. Indeed, those studies that employ a range of parameters and report prevalence still all identify groups who reliably do not perceive the ZT (Qi et al., 2022; Mohan et al., 2020).

The idea that ZT perception is, largely, binary (a person can be either ZT^+^ or ZT^−^) is also somewhat supported by individual data. In order to highlight this we make use of signal detection theory (Macmillan NA, 2002). Signal detection theory is a framework for separating out the sensitivity of a listener to a given signal (e.g., a real or Zwicker tone) from their bias to respond a given way (Macmillan NA, 2002). For example, we often present a signal (e.g., a pure tone) at a given intensity (e.g., sound level) a set number of times and calculate the proportion of times the participant correctly detects the signal, this is known as the “hit rate.” This measure, however, presents a problem: the participant might respond “yes I heard the signal” on every trial, i.e., they are biased toward reporting that they detected a signal. These individuals cannot be considered particularly good at the task but based on hit rate alone they appear to be very sensitive to the stimuli. To account for this, we include stimuli both with and without a signal (positive and negative stimuli, respectively). The proportion of trials a participant indicates the presence of a signal in response to a negative stimulus is called the “false alarm rate.” Some participants might consistently respond “yes” (regardless of the stimulus), they will have both a high hit and false-alarm rate. Conversely, some participants might have a moderate hit rate but a very low false alarm rate: these people detect the signal less frequently but, crucially, rarely identify a negative stimulus as containing a signal. Based on hit rate we could, wrongly, conclude the former participants were very good, when they are just biased toward respond “yes,” at the task and the latter not very good, when in fact they are they are just biased toward responding “no.”

Signal detection theory offers a method for reducing the influence of a person’s bias to measure a person’s sensitivity to a given stimulus using the d prime (*d’*) statistic. We calculate *d’* by taking the difference between hit rate and false alarm rate (as z scores): a low to moderate *d’* (e.g., 1–2 or above) is considered relatively sensitive and a value below this relatively insensitive. As well as sensitivity, we can use signal detection theory to measure decision criterion, which is independent of the actual sensitivity to the stimuli, rather reflecting an individual’s bias to a certain response (Macmillan NA, 2002).

**Figure 5:**
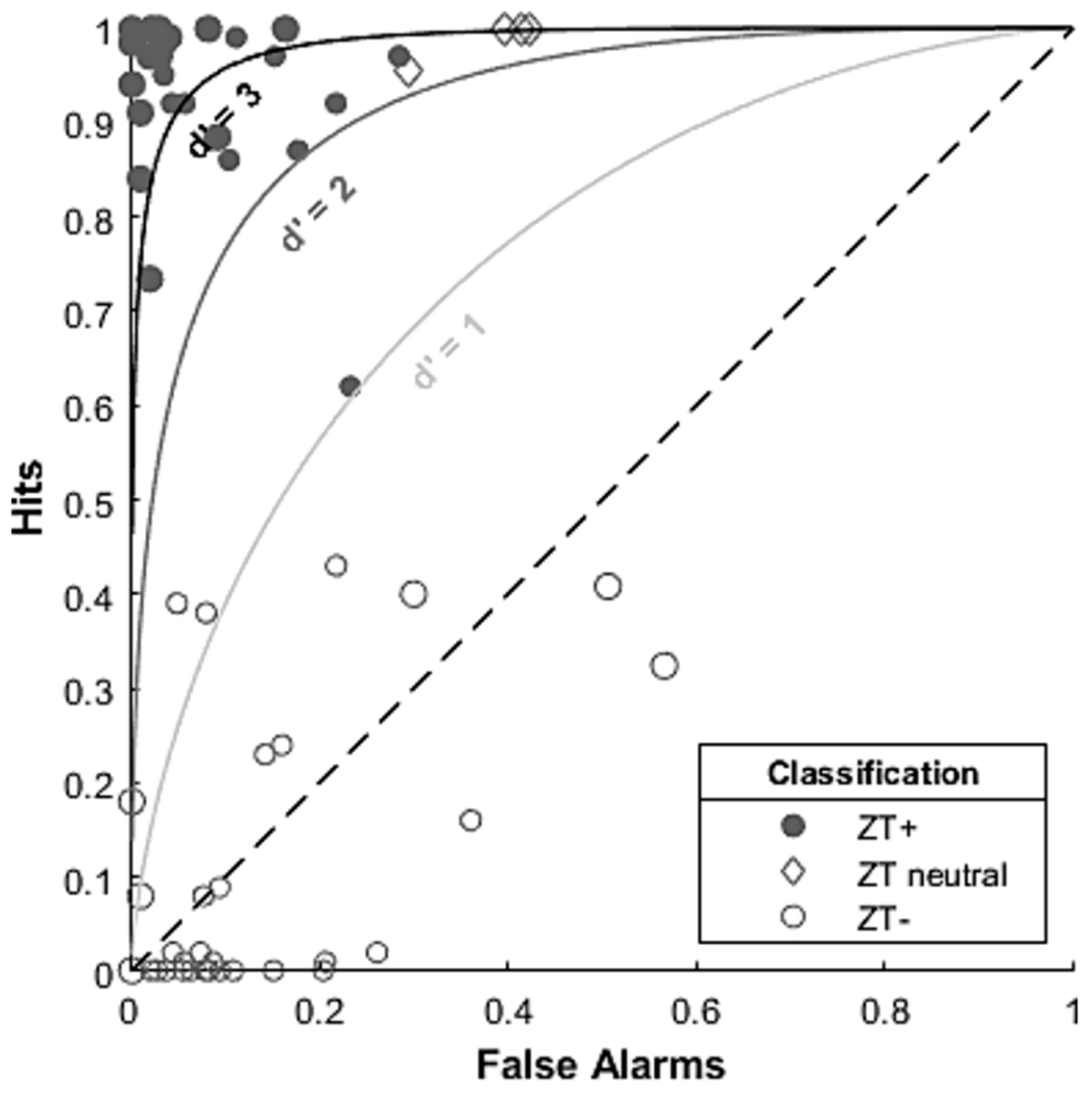
ROC plot demonstrating individual performance of participants in a small sample of ZT studies. Diagonal dashed line indicates chance performance. Curved lines indicated d prime values of 1, 2 and 3 indicating comparable sensitivity along these lines (according to signal detection theory). Data from: Chen et al. (2025) (small symbols) and DeGuzman (2012) (large symbols).

Most studies use a non-ZT inducing sound (wideband noise or non-ZT inducing notch noise) as a control stimulus to check for false alarms and so we can, where data is available, apply signal detection theory to these data. Two of these studies (DeGuzman, 2012; Chen et al., 2025) reported individual hit and false alarm scores, allowing us to retroactively calculate *d’,* and plot individual data points on a receiver operating characteristic plot (Figure 5). The vast majority of participants fell into two clusters (Cluster 1 (top-left), close to: hits = 1, false alarms = 0; Cluster 2 (bottom-left), close to: hits = 0 false alarms = 0) suggesting that on the whole most participants fall into one of the two groups, Cluster 1 comprising ZT^+^ individuals and Cluster 2 comprising ZT^−^ [while Chen et al. (2025) used a selection criterion that might skew there data into two groups, there is no evidence DeGuzman (2012) does the same]. These data also suggest that, on the whole, the classification criteria used (while different for these two studies), did a reasonable job of separating individuals into ZT^+^ and ZT^−^ groups. However, Figure 5 also demonstrates the difficulty in using criteria (based on hits and false alarms) to exclude/include participants. Participants grouped as “threshold” by DeGuzman (2012) (grey diamonds) showed similar sensitivity (e.g., *d’* > 2) to participants categorised as ZT^+^. Conversely, Chen et al. (2025) classify a participant with moderate sensitivity (*d’* ~ = 1, small filled circle on *d’* = 1 line) as ZT^+^ but two others with moderate sensitivity as ZT^−^ (small empty circles around 0.4 hits and above d’ = 1 line). We propose that it may be more suitable to use either a *d’* based threshold to group people into ZT^+^ and ZT^−^ groups or a statistical threshold based on single trial data to reduce the occasional inconsistencies in categorisation that might occur.

The question of whether Zwicker tone perception is binary or on a continuum remains open. A range of stimulus parameters can modulate whether a Zwicker tone will be perceived. For example, the best notch for inducing ZT perception is close to 4 kHz with an ~1 octave notch (see “Induction Stimuli” section). Increasing the notch width from 0.6 to 1 octave (notch centred on 4 kHz) increases the probability of detecting a ZT (Qi et al., 2022). Lowering the centre frequency of the notch (but keeping it at 1 octave) reduces the probability of detecting a ZT (Chen et al., 2025). In addition, the duration of the inducing stimuli may also alter the probability of detecting a ZT (see “Prevalence” section). Finally, given altering sound level modulates the frequency of the percept and the likelihood a tone will be detected in the frequency range of the ZT it also seems likely it will modulate the likelihood a ZT is perceived. While the current, though hardly extensive, data suggests ZT perception is binary rather than on a continuum studies employing a range of stimulus parameters (mapping out a large range of parameters) would be required in order to determine whether a person labelled as ZT^−^ is truly unable to hear ZTs under any circumstance or merely hears them over a restricted range of parameters.

### Threshold changes

ZT induction stimuli are associated with improved detection thresholds of subsequently presented tones in silence, in comparison to both broadband noise (Norena et al., 2000) and silence (Wiegrebe et al., 1996). Surprisingly, Wiegrebe et al. (1996) reported improvements are greater vs. silence than broadband noise, though this may be due to the different methodological approaches used to establish either threshold: Békésy tracking system for silence (Wiegrebe et al., 1996) and a 2-alternative forced choice paradigm for broadband noise (Wiegrebe et al., 1996; Norena et al., 2000).

Changes in detection threshold following notched noise are also reported outside of the ZT literature. The finding that notched noise presentation can improve signal detection of threshold vs. silence has been reported in animals exposed to long term notched noise, with improvements again seen within the frequency range of the notch and not outside (Krauss and Tziridis, 2021). Interestingly the authors also report that 91% of animals exposed to notched noise also developed tinnitus like behaviours at the frequency of the notch. Of those animals demonstrating tinnitus behaviour, 40% recovered post noise exposure when their hearing essentially experienced full recovery. Improvements vs. broadband noise are also confirmed in the literature (Alves- Pinto and Lopez- Poveda, 2008; Larsby and Arlinger, 1998), however the finding that narrower notches result in greater threshold reduction vs. silence is reportedly not the case vs. broadband noise, where wider notches see greater improvements (Larsby and Arlinger, 1998). The report that threshold improvement is greater at louder noise presentation (Wiegrebe et al., 1996) — at least for 30 dB vs. 40 dB - has been explored elsewhere and found to peak at 70 dB, reducing either side towards 50 dB and 90 dB (Alves- Pinto and Lopez- Poveda, 2008).

The finding that the frequency of the ZT is closely related to the frequency of greatest detection threshold improvement (Wiegrebe et al., 1996) has not been further confirmed within the literature due to the lack of ZT-frequency matching in other studies that measure threshold change across a range of frequencies (Norena et al., 2000; Alves- Pinto and Lopez- Poveda, 2008). On the basis of our plotting the ratio of percept to lower edge frequency as a function of presentation level (Figure 4), we can estimate the frequency of a ZT that might hypothetically arise following the notched noise presentation and compare this with reported frequencies of greatest threshold change. Norena et al. (2000) presented a notch with a lower edge frequency of 3.4 kHz at 40 dB SPL and subsequently we would predict the frequency of greatest threshold change to be 3.95 kHz. Indeed, they find the greatest improvement at 4 kHz, the tested frequency closest to the prediction. Meanwhile, Alves- Pinto and Lopez- Poveda (2008) utilise a notch with lower edge frequency 7 kHz ranging between 50 and 90 dB SPL presentation level. At 50 dB we would predict the frequency of greatest change to be 8.46 kHz and Alves-Pinto report 8.5 kHz. This comparison of reported values versus prediction can be seen in Figure 4. At higher presentation levels (70 dB+) our predictions are less accurate, consistently returning higher frequencies than are reported. However, it should be noted that a relatively narrow notch is used, between 7 and 9 kHz, and at 70 dB + we would predict the frequency of greatest change to be within the upper band of noise, which may explain the reduced reliability, at least in part.

### Mechanism

A number of mechanisms have been proposed to account for the generation of the ZT percept including habituation (Fastl et al., 2001), gain adaptation (Parra and Pearlmutter, 2007) and stochastic resonance (Schilling et al., 2021). Where authors have presented a potential mechanism, they often evoke lateral inhibition to some extent, a process whose involvement in ZT generation was first proposed by Norena et al. (2000). It should perhaps be noted that the following mechanisms need not be mutually exclusive.

#### Lateral inhibition

**Figure 6:**
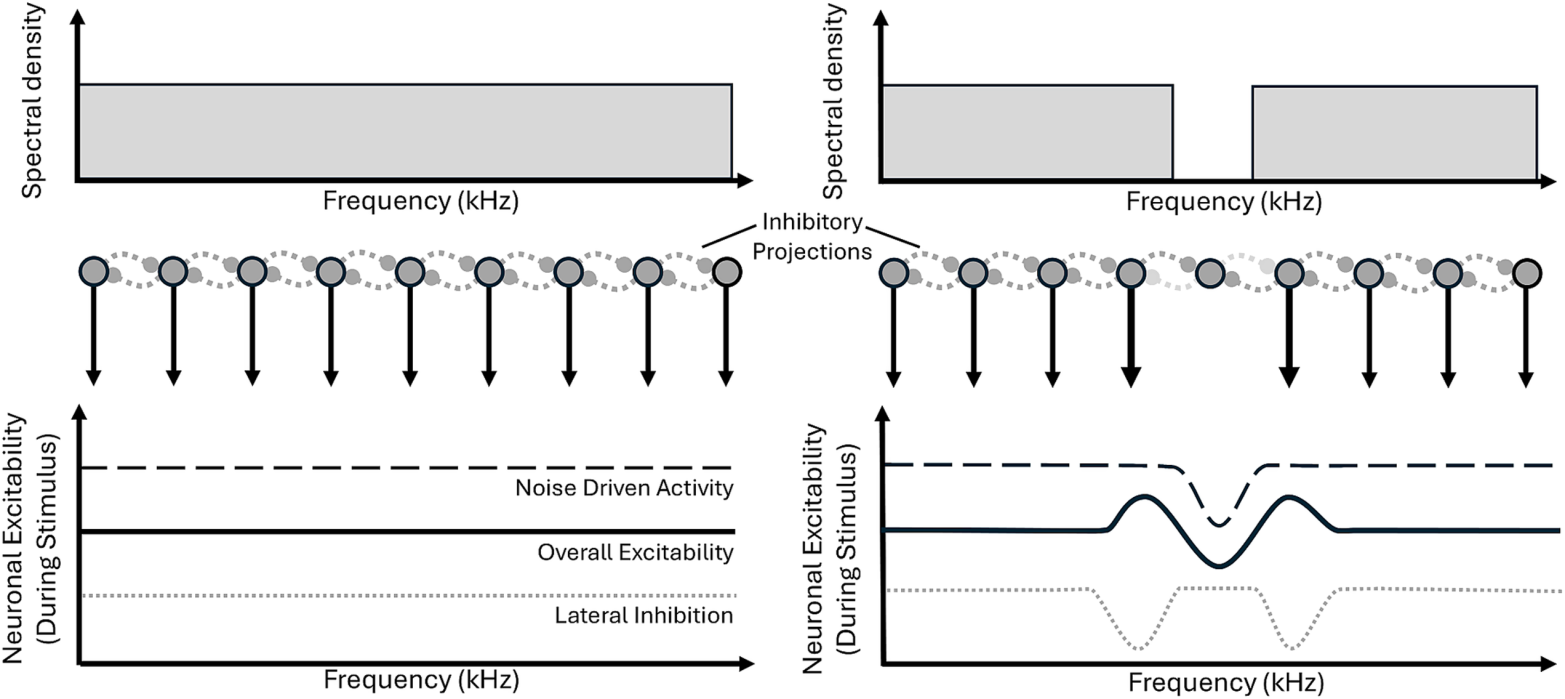
Lateral inhibition model proposed by Norena et al. (2000). Broadband noise (bottom left), when fed through a neural model of lateral inhibition (middle left: dark gray circles with black surround represent neurons, dashed lateral lines indicate inhibitory connections), produces relatively even excitability of neurons (top panel, black line), across frequency. Whereas, notched noise presentation (top right) results in decreased excitability of neurons within the notch (middle: absent downward arrow from central neuron, top panel: dip in dashed line) in turn reducing lateral inhibition (middle: light gray horizontal connections from center neuron, bottom: two dips in the dotted line) to the noise edge frequencies, which enhancing excitability to the edge frequencies (bottom: solid line). This means within the notch, overall excitability (bottom: solid line) is reduced, below the baseline level, due to lateral inhibition (from the strongly excited edge frequencies).

Lateral inhibition is the process by which one neuron, via a lateral projection, inhibits a neighbouring neuron. Within the context of the tonotopically organised central auditory system (Bourk et al., 1981; Guinan et al., 1972; Humphries et al., 2010; Imig and Morel, 1985; Morel et al., 1993; Stiebler and Ehret, 1985) these neighbours are neural populations tuned to similar frequencies. For a notched noise, sound driven neurons (i.e., those tuned to the noise) will inhibit non-driven neurons (those tuned to the notch). Furthermore, with lateral inhibition an increase in neural activity in frequency channels around the edges of the notch would be anticipated as they receive reduced inhibition from within the notch due to the lack of driven activity (Figure 6).

Lateral inhibition is often invoked because it can be used to explain patterns in imbalances of excitation and inhibition that may give rise to the ZT and associated threshold changes (we will refer to these in greater detail later on). It should be noted that alternate mechanisms, such as surround inhibition (Lakunina et al., 2020), might also achieve the same affect however, for ease, we will use the term lateral inhibition going forwards simply to refer to a situation in which activity in a frequency channel results in a net-inhibitory effect on the activity of a neuron in a different channel.

#### Habituation

**Figure 7:**
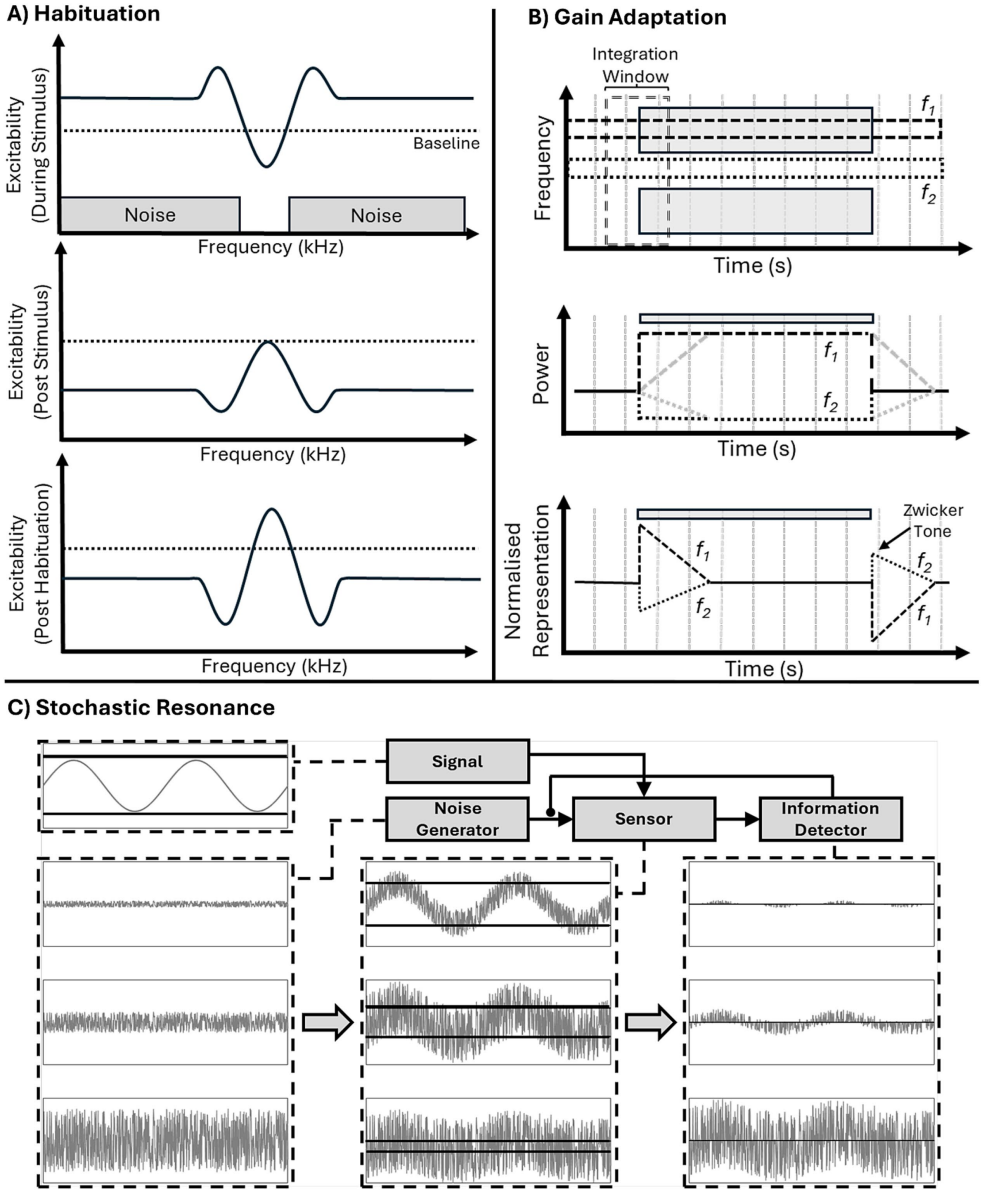
Three proposed mechanisms of ZT generation. **(A)** Habituation: during notch noise stimulation (top) excitability is decreased within the notch and increased at notch edge (as in the lateral inhibition model). Post stimulus (middle), habituation leads to decreases in excitability in previously active frequency ranges, but not within the notch. Post habituation lateral inhibition (bottom) the habituated noise edge frequencies produce a reduction in lateral inhibition and, hence, increased excitability (above baseline) within notch frequency range. **(B)** Gain adaptation: schematic (top), showing two frequency bands, f_1_ and f_2_, in the noise and notch regions, respectively, and a temporal integration window (over which long-term power will be calculated). At noise onset instantaneous power in the f_1_ band immediately increases (dark lines), whereas, average long-term power (gray dashed line) increases gradually (after sound onset) and takes time to plateau. Instantaneous power is divided by long-term power (i.e., the gain) to produce output power, this means at sound onset there will be a brief period of high output power before the long-term power plateaus and reduces gain (bottom, dashed black line). Likewise, at sound offset long-term power remains high for a period of time (middle: dashed grey line), meaning that it is much greater than instantaneous power (middle: dashed black line), resulting in a period of decreased output power (relative to background activity). Lateral inhibition inverts this process in the f_2_ band leading to a decrease in both instantaneous (middle: dotted black line) and long-term power (middle: dotted gray line) but the same temporal imbalance. Therefore, at sound onset there is a brief decrease in power in the f_2_ band and, crucially, a brief increase in power at sound offset leading to perception of a Zwicker tone (bottom: dotted line). **(C)** Stochastic Resonance A signal (top-left) is of insufficient amplitude to cross the detection threshold (dark horizontal lines). Addition of different levels of noise (first column) by the noise generator to the sensor, allows signal activity to cross the detection threshold (second column). Resultant activity is received by the information detector (third column), which modulates noise input to allow for ideal signal communication. Too little noise (top row), and insufficient signal is communicated, too much (bottom row) and the signal is lost in the noise.

Habituation, refers to a neuron reducing its excitability following frequent stimulation and has been proposed as a potential mechanism to explain ZT perception (Fastl et al., 2001). During stimulation, channels corresponding to the frequencies just inside the noise edges will be most active, being driven by the noise while not receiving lateral inhibition from within the notch (Figure 7A, top panel), and in turn will undergo greatest habituation, seeing their excitability (and spontaneous activity) most greatly reduced (Figure 7A, middle panel). At stimulus offset they will demonstrate the lowest spontaneous activity and as such will exert very little lateral inhibition into the un-habituated notch, resulting in an increase in spontaneous activity in those neurons tuned to notch-frequencies (Figure 7A, bottom panel). This increase in spontaneous activity could then be interpreted as a sound, resulting in ZT perception.

#### Gain adaptation

Parra and Pearlmutter (2007) suggested a process of gain adaptation, in which the instantaneous power in individual frequency channels is divided by average power, calculated over a integration window (Figure 7B, top panel), in turn decreasing gain following periods of high power and increasing after periods of low power. This rescaling of power, in combination with lateral inhibition, results in channels within the notch, with reduced long term average power due to being maximally inhibited (Figure 7B, middle panel), subsequently increasing their gain to a relative increase in power, potentially resulting in illusory perception due to the increased normalised representation of spontaneous activity (Figure 7B, bottom panel). The temporal integration window of gain adaptation, integrating recent power over a certain time frame, also provides an explanation for the increase, and eventual plateau, of the percept duration as a function of the inducing noise duration (Lummis and Guttman, 1972). Increasing the duration of the noise increases the proportion of time there is power within this window (i.e., increases average power), until the duration of the noise reaches the length of the window (at which point there power throughout the entire window).

#### Stochastic resonance

A more recent mechanistic explanation is the stochastic resonance interpretation (Schilling et al., 2021). In this model the detection of low-level signals can be improved by the addition of low-level noise. A hypothetical feedback loop is proposed consisting of a ‘sensor’, ‘information detector’, and ‘noise generator’ (Krauss et al., 2016). The noise generator feeds noise to the sensor - in the form of increased spontaneous activity - modulated by the information detector which receives input signals from the sensor. In turn, the information detector can control the addition of noise to the sensor, based on the detection of signals in the incoming stream, in order to maximise transmission (Figure 7C). In the case of ZT-inducing stimuli, the notch frequencies would be interpreted as deafferented input and would be the recipient of additional noise. ZT induction is then a result of the subsequent increased spontaneous activity being perceived as a tone. While the above model is more abstract and computational in nature, it has been suggested that the source of noise may be the somatosensory system (Schilling et al., 2021; Krauss et al., 2016), citing the modulation of the tinnitus percept by jaw movements as evidence.

#### Modified lateral inhibition: asymmetric inhibition

While the above theories account for an increase in intrinsic excitability and subsequent spontaneous activity which can be misinterpreted by the brain as a sound, they do not explain all the qualities of the ZT. For example, the relationship between ZT pitch and lower-edge frequency (Figure 4) and the lack of secondary percept arising at the upper-edge frequency. Accordingly, the lateral inhibition model (Norena et al., 2000) has since been further developed to account for this by introducing asymmetric lateral inhibition, in which lower frequencies exert a greater inhibitory effect over relatively higher frequency channels (Fastl et al., 2001). Asymmetric inhibition has been observed within the auditory system in animal models (Gilday et al., 2023; Catz and Noreña, 2013; Zhang et al., 2003), and inferred from a human imaging study (Okamoto et al., 2007) and two-tone suppression studies (Penner, 1980; Shannon, 1976). However, while the imaging study provides a congruous interpretation of greater inhibition by lower frequencies over higher, two-tone suppression data would suggest that, at least at low-moderate sound levels, the higher frequency range exerts a greater inhibitory effect (Penner, 1980; Shannon, 1976). Similarly, animal studies are inconsistent reporting skews in both directions (Gilday et al., 2023; Catz and Noreña, 2013), as well as suggesting this bias may vary with frequency (Zhang et al., 2003). As such the role of asymmetric inhibition in ZT generation is less clear than perhaps it first seems.

#### Modified lateral inhibition: noise-detection neurons

**Figure 8:**
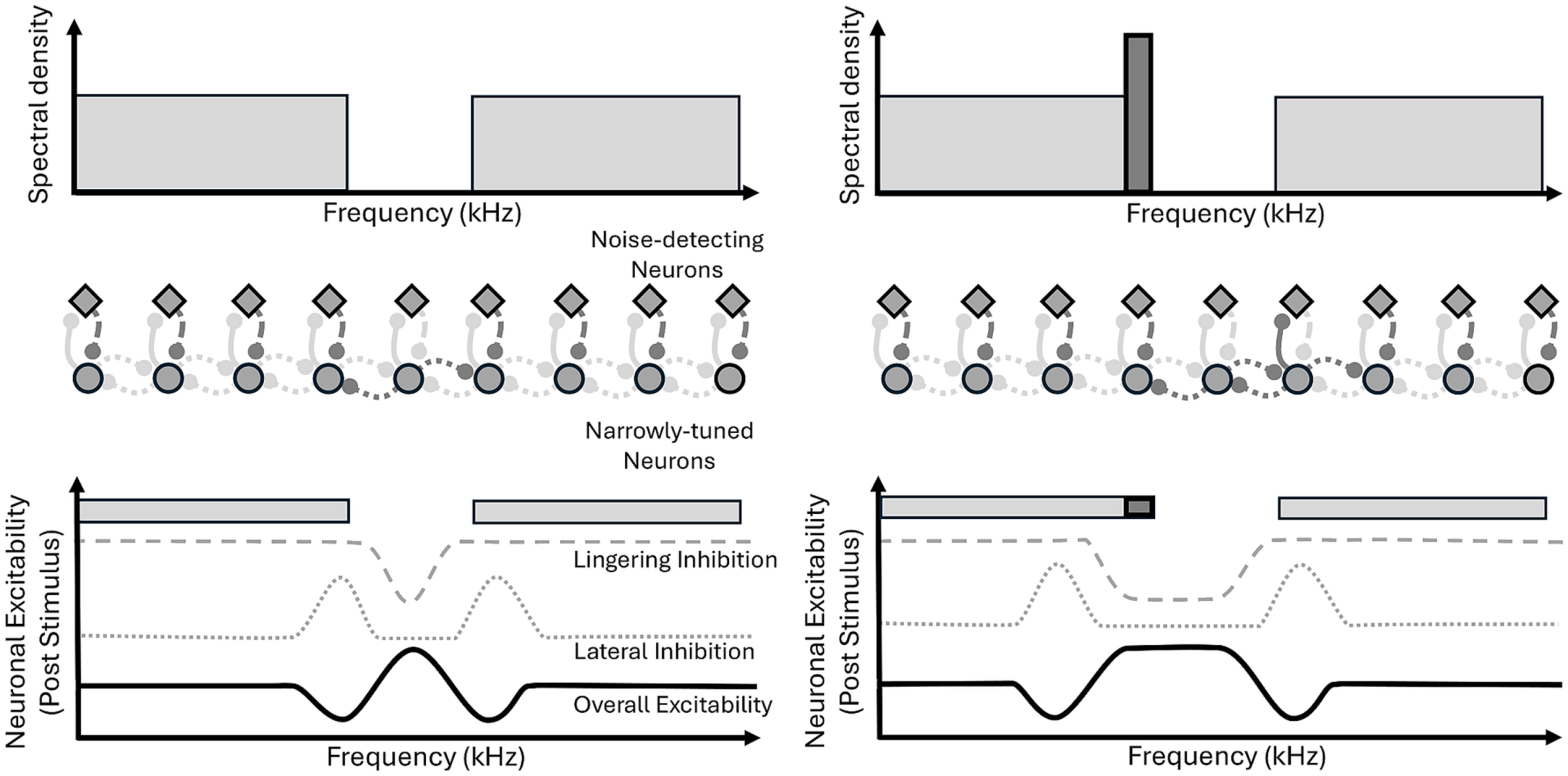
Modified lateral inhibition model. Notched noise presentation drives activity in noise-detecting neurons (diamonds), except within the undriven, notch frequency range. Post stimulus, noise-detecting neurons exert lingering inhibition (dashed line) over previously driven frequency ranges; lateral inhibition (dotted line) is greatest at notch edges, driven by notch frequency ranges free from lingering inhibition. Overall excitability (solid line) is greatest in the notch frequency range, free from both forms of inhibition, and least at the notch edges which are inhibited by both lateral and lingering inhibition. During presentation of notched noise with an embedded pure tone, highly driven narrowly-tuned neurons (circles) inhibit noise-detecting neurons in the frequency channel of the tone. In turn, the range of lingering inhibition no longer includes the frequencies surrounding the tone, and lateral inhibition also shifts to a lower frequency, resulting in an extension in the range of greatest excitability.

Notched stimuli with tones embedded at the lower edge frequency (Figure 8, bottom right) produce a ZT pitch below the frequency of the tone and within the frequency range of the noise, this has led to modification of the lateral inhibition model, which we will refer to as “modified lateral inhibition” (Franosch et al., 2003; Fastl et al., 2001). Slow-responding, inhibitory ‘noise detecting neurons’ have been suggested as an additional component to the model to help explain this particular example of ZT generation (Figure 8, diamonds in the middle panel). Their slow dynamics, allow them to exert a lingering inhibitory effect post stimulus offset, resulting in a reduced activity within the noise frequencies. This results in disinhibition of the notch frequencies, via reduced lateral inhibition. Furthermore, it is proposed that these noise-detecting neurons are inhibited by more narrowly tuned neurons (Figure 8, circles in the middle panel), such that the presence of a pure tone, which has much greater spectral density than wideband noise, inhibits the noise-detecting neuron activity in the associated frequency channel. Subsequently, the lingering inhibition that is exerted by the ‘noise detecting neurons’ is not seen at the frequency of the pure tone, and the region of uninhibited, increased activity is extended to encompass a range of frequencies surrounding the tone, including below the lower-edge frequency, surrounding the tone frequency (Figure 8, top right). In turn, the edge of lingering inhibition has shifted, and this now represents the lower edge frequency, hence the ZT is generated below the tone frequency.

#### Offset responses

While lateral inhibition can be used to explain the frequency range of excitatory-inhibitory (EI) imbalance, it does not itself represent a neural correlate of the ZT percept. Hence, lateral inhibition requires a process to convert inhibition into increases in activity, of which the above mechanisms are an example.

Alternatively, it could be proposed that sustained lateral inhibition (caused by long duration or repeated notch-noise), which strongly inhibits the cells within the notch, might lead to a post-inhibitory rebound, i.e., excitatory, firing at sound offset (Kopp-Scheinpflug et al., 2018). This excitatory response in neurons tuned to the notch might then be interpreted as the arrival of a real sound. In the upper auditory system, such as the auditory cortex, sound offset responses are tuned close to but not at the same frequency as sound onset responses (Sollini et al., 2018), suggesting neurons in the notch should exhibit sound offset responses. Indeed, studies performed in animals have observed sound offset responses in auditory cortex following notched noise (Schilling et al., 2023) and increased spontaneous firing by auditory cortical neurons immediately following notched noise exposure vs. broadband noise (Norena and Eggermont, 2003) both of which being maximal in neurons tuned to frequencies within the notch.

Finally, it is important to highlight the fact that, while all of the above propositions allude to the neural correlates of the ZT, they do not make attempt to address the fact that it is not universally perceived. The strength of the mechanism, in generating a Zwicker tone, could modulate the probability that that neural correlate will give rise to an illusory tone percept but there are additional factors, which to date, have not been included in this consideration. One possible example from signal detection theory might be differences in criterion. It is entirely possible that differences in criterion drive differences in ZT perception, an aspect that the use of signal detection theory has the potential to capture.

#### Threshold changes

Another aspect to take into account when considering these proposed models is the aforementioned threshold effects. The increase in excitability and spontaneous activity associated with ZT generation in these models provides an effective explanation for the reduced thresholds for tones presented at the ZT frequency – indeed, the pitch of the ZT is likely associated with the frequency channel with the greatest increase in excitability and subsequently the greatest reduction in threshold. Furthermore, the lateral inhibition framework presented by Norena et al. (2000), also predicts that those channels corresponding to the edge frequencies of the notch would see the greatest activity during noise stimulation (Figure 6). All of the above mechanisms incorporate lateral inhibition and predict diminished activity at these points of greatest activity and in turn also provide a potential explanation for the associated increase in threshold seen at these frequencies (Figure 7).

Lateral inhibition may also begin to explain this relationship. Auditory filter widths have been shown to increase with increasing sound level (Moore and Glasberg, 1987), and likewise there is an increase in neural tuning widths, in both excitatory and inhibitory neurons, throughout the auditory system. This predicts that as both excitatory and inhibitory receptive fields widen, the extent beyond the edge of the lower edge frequency of the notch, of both would increase. In turn, the area of greatest inhibition would also move to higher frequencies, resulting in a ZT of higher pitch.

#### Cortical electrophysiology results

While above we discuss a potential increase in activity in the frequency range of the ZT, MEG imaging studies show that exposure to spectrally contrasted stimuli in the form of notch or comb-filtered noise leads to a subsequent reduction in N1m amplitude of AEF for tones of a frequency within the notch or stop band regions of the noise (Pantev et al., 2004; Okamoto et al., 2004; Okamoto et al., 2005). This effect is seen for combed noise including stop bands at frequencies not dissimilar to those that induce ZTs (0.5–2.8 kHz) (Pantev et al., 2004; Okamoto et al., 2004), however they are narrow bands, and the use of combed noise means we cannot distinguish the contribution of different frequency ranges. Meanwhile where a wider range of notches are used at a single centre frequency (1 kHz), the effect is only seen for the narrower notch (Okamoto et al., 2005). This effect is enhanced by increasing the noise level at frequencies surrounding the notch, though increasing the width of amplified frequencies can lead to a reduction in enhancement (Stein et al., 2013) – this may be due to an increase in activity over a greater range of frequencies resulting in greater inhibition of neurons tuned to the edge frequency, resulting in disinhibition of those neurons in the centre of the notch. These findings are in contradiction with DeGuzman (2012) who reported an increase in amplitude 140 ms after the presentation of ZT stimuli. Though this may be a reflection of the fact that participants had been sub-selected based on their ability to perceive the ZT, or alternatively the aforementioned differences in notch parameters employed. In addition, reduced N1m amplitude (Pantev et al., 2004; Okamoto et al., 2004; Okamoto et al., 2005) would perhaps be unexpected when considering the improvement in detection thresholds that are observed following notched noise, though this may again be a result of the differing noise parameters, indeed it is reported that narrower notches lead to reduced threshold improvement (Wiegrebe et al., 1996).

Unfortunately, due to the heterogeneity of results from cortical electrophysiology studies investigating the ZT, it is difficult to draw any conclusions as to how the observed correlates relate to the psychophysical findings surrounding the ZT with any real confidence.

#### Zwicker tones in animal models

When considering animal studies, we must consider the assumption that the models are equivalent and that the animals do indeed perceive the ZT. Schilling et al. (2023) have attempted to assess perception in gerbils using gap pre-pulse inhibition of the acoustic startle reflex (GPIAS) – often used in perception assessment in tinnitus models - as well as a conditioned GO-NOGO paradigm in which animals are trained to respond discriminately to white noise followed by silence vs. white noise followed by a pure tone. They report successful perception assessment via the GPIAS paradigm, with a 5 kHz centred notch decreasing the startle response vs. continuous white noise to a greater extent than silence following white noise. It should be noted that in tinnitus perception, a lesser decrease would be predicted as the tinnitus ‘fills in’ the gap in the noise (Turner et al., 2006), however in the case of ZT the authors argue that a tonal percept is not sufficiently similar to the preceding noise to ‘fill it in’. Instead, it presents an even more salient signal vs. silence to the oncoming startle pulse, resulting in a decreased startle response. The animals are unable to perform the GO-NOGO task in response to ZTs in place of pure tones, however only 2 kHz tones are used, while the GPIAS paradigm showed that 5 kHz were more effective. As it stands, further investigation is probably required to confirm the perception of ZT in animals. However, Qi et al. (2022) used Zwicker tones to create a mismatch negativity in humans and this approach might present a paradigm that could be successfully applied to animal models to assess illusory perception.

#### Zwicker tones and tinnitus

As previously alluded to, tinnitus and the ZT share a number of characteristic similarities, both being illusory percept’s associated with a spectral contrast (hearing loss and notch or low frequency band-pass noise, respectively) in input and both percepts falling within the unstimulated frequency region (for tonal tinnitus), i.e., in the notch for ZTs; often occurring within the range of the hearing loss for tinnitus.

Despite a number of papers drawing comparison between ZT and tinnitus, only one study has investigated ZT in people with tinnitus, though the reported finding that ZT perception is far more prevalent in tinnitus participants is a striking one (Parra and Pearlmutter, 2007). It should, however, be noted that the study reports no power calculation and the stimuli selected for the study are non-standard in comparison to the rest of the literature. While the range of notch-centre frequencies is not atypical, the use of a consistent 4 kHz notch width, rather than a notch scaled by octaves or ERBs results in relatively wide notches of up to 17.4 ERBs (see Figure 3 and Table 3 for comparison). This broad notch width introduces the possibility of a confound. Most people with tinnitus have a hearing-loss (Langguth et al., 2013) and most people with hearing-loss have broader auditory filters (Leek and Summers, 1993), this means, in general, auditory filters in a tinnitus ear are wider than that of a healthy ear. The absence of studies reporting a ZT percept at wide notch widths suggests that this may be a limiting factor on ZT perception (much like the notch being too narrow). It is possible that people with tinnitus, with wider auditory filter widths, might find ZTs easier to perceive with wider notch widths, when compared to controls. In addition, it is not reported to what extent participants reacted positively or negatively to each of the different stimuli, only that ZT^+^ individuals did not respond positively to white noise. ZT perception was assessed based on consistent reporting of “a perception of some form of ringing, however faint it might be” following any of the notched stimuli, but not broadband noise. The open-ended nature of this reporting could result in an overestimation of ZT perception in people with tinnitus if differences in false alarms existed, though both groups – normal hearing and tinnitus – were provided with the same prompt. Furthermore, tinnitus was self-reported, assessed on the basis of reporting “spurious ringing on a regular basis,” and no tinnitus assessment questionnaires of any form were employed. With these factors considered we believe further investigation into ZT prevalence in people with tinnitus may prove useful to tinnitus researchers that wish to employ the ZT to better understand its relationship to tinnitus.

Alternative supporting evidence has also been proposed here, such as improved hearing thresholds following notched noise being related to the finding that people with tinnitus tend to have lower hearing thresholds than those without (Schilling et al., 2021; Krauss et al., 2016; Gollnast et al., 2017). However, while in children under the age of 18 this is seen even at higher frequencies, in the general population the effect is only seen at 3 kHz or below, while the frequency of the tinnitus percept tends to lie at higher frequencies. While both of these phenomena have been observed in ZT^+^ populations, with particular reference to the ZT frequency, threshold changes are also reported in populations not screened for ZT perception – and in turn presumably consisting of a mix of ZT^+^ and ZT^−^ subjects. As such, should ZT perception and tinnitus be related, it is difficult to directly associate ZT perception with these threshold effects. In turn, it may be fruitful to investigate these effects, comparing between those subjects who can and cannot perceive ZT.

If we are to make the assumption for the time being that tinnitus and ZT perception are indeed linked in some way, we next have to ask ‘how?’ We propose 3 routes by which this relationship might be explained: (1) Common mechanistic induction: It is clear that notched-noise stimuli can reliably induce the Zwicker tone percept with a high degree of discriminability from control stimuli, such as narrow notch or wideband noise. Therefore, it seems likely notched-noise produces neural activity that mimics that of a pure tone. This is supported by the animal literature that demonstrates increased transient activity, referred to as an offset-response, at the end of the notched noise and in neurons tuned to the notch frequency (Schilling et al., 2023). One possibility is that the mechanism that produces this activity, e.g., lateral inhibition (Lummis and Guttman, 1972; Norena et al., 2000) or stochastic resonance (Schilling et al., 2021), could play a role in producing the tinnitus percept. Parra and Pearlmutter, when presenting their gain adaptation mechanism hypothesis suggest that impaired outer hair-cell function and amplification of faint sounds in people with tinnitus may lead to an increased reliance on central gain to manage dynamic range. This may potentially exacerbate the degree to which spontaneous activity is increased, resulting in greater likelihood of ZT perception (Parra and Pearlmutter, 2007). (2) Increased probability of misinterpreting illusory neural correlates: It is also possible that people with tinnitus are more likely to interpret the evoked neural activity, in which ever form, into a perceptual object, e.g., due to a compromised limbic system (Rauschecker et al., 2010). This could be due to differences in a range of factors from cognitive processes, such as: prediction (Hullfish et al., 2019) or attention (Roberts et al., 2013), or a sensory process such as differences in sensory criterion (Macmillan NA, 2002). (3) Tinnitus related changes increase the probability of Zwicker tone prediction. Tinnitus is most commonly caused by hearing-loss (Langguth et al., 2013) which is also commonly associated with broader auditory filter widths (Leek and Summers, 1993). In the Parra and Pearlmutter (2007) the notch widths used were much wider than those used in studies using normal hearing listeners, potentially meaning this stimuli may be more likely to induce ZT perception in participants with broader auditory filter widths.

Finally, when considering the potential relationship between tinnitus and ZT perception, it is worth bearing in mind that, in the way that not all people can perceive the ZT, not all people with hearing loss (of which notched/spectrally contrasted noise can be considered a transient model) go on to develop tinnitus. If they are indeed related, exploring what defines an individual’s ability to perceive the ZT may aid in our understanding of tinnitus, and may well potentially identify the ZT as a tool for screening an individual’s likelihood of developing the condition.

## Conclusion

ZT perception has been reliably reproduced over a wide range of studies, however, the prevalence of ZT perception is highly variable and depends on a range of factors, such as the criteria used to categorise people into ZT^+^ and ZT^−^ groups, the instructions given to the participants (Zwicker, 1964) and, crucially, the parameters used for the inducing noise (see Figure 3; Tables 2, 3). While we know many of the parameters that can produce a Zwicker tone it is not fully known where the bounds of these parameters fall, i.e., the parameters where a ZT is no longer perceived, and so it seems reasonable to assert that filling in these gaps in our knowledge might improve our understanding of how ZTs are generated. The same parameters that produce a ZT percept also reduce auditory thresholds of tones presented around the time of the ZT percept (e.g., estimate ZT pitch in Figure 4). This strongly hints toward the idea that ZT inducing stimuli induce activity in neurons that are tuned to this frequency, an idea supported by the animal literature (Schilling et al., 2023). Though the exact mechanism that creates this activity and where in the brain it originates is still a matter of debate. Finally, beyond the obvious similarities between tinnitus and ZTs, to date, only one study has demonstrated an association, i.e., that ZT prevalence appears to be significantly higher in people with tinnitus. This suggests a replication of this result, while potentially addressing any potential confounds of this study, would be warranted.

If, long-term, the link between tinnitus and ZTs is proven then ZTs could aid our understanding of tinnitus and, perhaps, even be used clinically. While speculative we will try to highlight the possible uses of ZT based tasks. For example, if ZT perception is higher in people with tinnitus it is logical to ask is: why? Assuming this represents a causal relationship, it could be that people who can hear ZTs are at a higher risk of developing tinnitus (and therefore ZT perception could be a useful screening tool for tinnitus). Alternatively, it might be that the development of tinnitus increases the likelihood of ZT perception, in which case understanding the generation of ZTs might elucidate the changes that occur in people with tinnitus. In either case, if a causal relationship exists then understanding this relationship would improve our understanding of tinnitus. ZT induction allows tight experimental control over: whether an illusion is perceived, when it perceived, how strong the percept is, how long it lasts and the homogeneity of the parameters used to create it. As such, from a research perspective, it offers many advantages over tinnitus as a model for understanding the mechanisms that lead to the generation of illusory sound perception. In addition, by creating illusions in non-clinical populations (free from the significant stress/anxiety and concomitant conditions chronic tinnitus can cause) ZTs might be a useful tool to understand the cognitive factors that influence the perception of illusory sounds.
